# Differential associations of dietary patterns with estimated 10-year cardiovascular risk in diabetes subtypes

**DOI:** 10.1038/s41598-026-57026-y

**Published:** 2026-07-06

**Authors:** Katharina S. Weber, Sabrina Schlesinger, Alexander Lang, Janina Goletzke, Cara Övermöhle, Eike A. Strathmann, Klaus Straßburger, Nitika Singh, Oana-Patricia Zaharia, Sandra Trenkamp, Robert Wagner, Wolfgang Lieb, Anette E. Buyken, Michael Roden, Christian Herder, GDS group, Robert Wagner, Michael Roden, Christian Herder, H. Al-Hasani, B. Belgardt, G.J. Bönhof, G. Geerling, A. Icks, K. Jandeleit-Dahm, O. Kuß, E. Lammert, S. Schlesinger, V. Schrauwen-Hinderling, J. Szendroedi, Sandra Trenkamp

**Affiliations:** 1 Institute of Epidemiology, Kiel University, Niemannsweg 11, D 24105 Kiel, Germany; 2 Institute for Biometrics and Epidemiology, German Diabetes Center, Leibniz Center for Diabetes Research at Heinrich Heine University Düsseldorf, Düsseldorf, Germany; 3 German Center for Diabetes Research (DZD), Partner Düsseldorf, München-Neuherberg, Germany; 4 Institute for Clinical Diabetology, German Diabetes Center, Leibniz Center for Diabetes Research at Heinrich Heine University Düsseldorf, Düsseldorf, Germany; 5 Faculty of Natural Sciences, Institute of Nutrition, Consumption and Health, Paderborn University, Paderborn, Germany; 6 Department of Endocrinology and Diabetology, Medical Faculty and University Hospital Düsseldorf, Heinrich Heine University Düsseldorf, Düsseldorf, Germany; 7 Institute for Clinical Biochemistry and Pathobiochemistry, German Diabetes Center, Leibniz Center for DiabetesResearch at Heinrich Heine University Düsseldorf, Düsseldorf, Germany; 8 Institute for Vascular and Islet Cell Biology, German Diabetes Center, Leibniz Center for Diabetes Research at Heinrich Heine University Düsseldorf, Düsseldorf, Germany; 9 Department of Ophthalmology, Medical Faculty and University Hospital Düsseldorf, Heinrich Heine University Düsseldorf, Düsseldorf, Germany; 10 Institute for Health Services Research and Health Economics, Centre for Health and Society, Faculty of Medicine, Heinrich Heine University Düsseldorf, Düsseldorf, Germany; 11 Institute for Health Services Research and Health Economics, German Diabetes Center, Leibniz Center for Diabetes Research at the Heinrich Heine University Düsseldorf, Düsseldorf, Germany; 12 Department of Diabetes, Central Clinical School, Monash University, Melbourne, Australia; 13 Centre for Health and Society, Medical Faculty and University Hospital Düsseldorf, Heinrich Heine UniversityDüsseldorf, Düsseldorf, Germany; 14 Institute of Metabolic Physiology, Faculty of Mathematics and Natural Sciences, Heinrich Heine University Düsseldorf, Düsseldorf, Germany; 15 Department of Endocrinology, Diabetology, Metabolic Diseases and Clinical Chemistry (Internal Medicine I), University Hospital of Heidelberg, Heidelberg, Germany; 16 German Center for Diabetes Research (DZD), Neuherberg, Germany; 17 Joint Heidelberg-IDC Translational Diabetes Program, Helmholtz Center Munich, Neuherberg, Germany

**Keywords:** Dietary fiber, Mediterranean diet, DASH diet, Plant-based diet, Diabetes clusters, Diabetes-related complications, Cardiology, Diseases, Endocrinology, Health care, Medical research, Risk factors

## Abstract

Diet impacts cardiovascular disease risk, but the association across diabetes subtypes is unclear. We thus aimed to assess the association between dietary factors and estimated 10-year cardiovascular disease risk across diabetes subtypes in the German Diabetes Study (GDS). Participants with recent-onset diabetes and from the 5-year follow-up examination were combined (n=635, mean age 47.5±12.9 years, 60% male) and classified into diabetes subtypes. Subtypes with prevalence >10% were included in cross-sectional analyses: severe autoimmune diabetes (39%), mild obesity-related diabetes (31%), and mild age-related diabetes (30%). Carbohydrate quality parameters and adherence to dietary patterns were assessed from food frequency questionnaires. Differences in associations between these dietary factors and 10-year cardiovascular risk estimated by SCORE2-Diabetes across subtypes were examined using multivariable-adjusted linear regression models including an interaction term. In people with severe autoimmune diabetes, the overall and the healthful plant-based diet index were associated with lower estimated 10-year cardiovascular risk (ß [95% CI] -6.8 [-12.1; -1.2] and -8.8% [-14.0; -3.2] per 1-standard deviation increases, respectively), while these associations were not evident among the remaining two subtypes (p-value for interaction between subtypes <0.05). Our findings suggest subtype-specific associations of plant-based dietary patterns with estimated cardiovascular risk, warranting further research from longitudinal and intervention studies. **Trial registration:** Clinicaltrials.gov: NCT01055093

**Supplementary Information:**

The online version contains supplementary material available at 10.1038/s41598-026-57026-y.

## Introduction

Cardiovascular diseases (CVD) are a major cause of morbidity and mortality in Europe^1^. CVD-related morbidity and mortality are considerably higher in people with diabetes than in those without^2^. Diabetes itself is an independent CVD risk factor. Furthermore, people with diabetes tend to accumulate major CVD risk factors more commonly as compared to the general population, further increasing their CVD risk^3^.

Mounting evidence suggests that the disease progression and risk for complications, including CVD, vary among people with diabetes. In this context, five diabetes subtypes have been described: severe autoimmune diabetes (SAID; mainly corresponds to type 1 diabetes) and four subtypes formerly uniformly classified as type 2 diabetes, namely severe insulin-deficient diabetes (SIDD), severe insulin-resistant diabetes (SIRD), mild obesity-related diabetes (MOD), and mild age-related diabetes (MARD)^4–6^.

Diet is crucial in modifying CVD risk in people with diabetes^3^. The Mediterranean diet and the Dietary Approaches to Stop Hypertension (DASH) diet are known to reduce CVD risk^7,8^. Increasing evidence also suggests benefits of a plant-based diet in people with diabetes^9^. Furthermore, a diet low in glycemic index/glycemic load and high in dietary fiber has been associated with reduced CVD incidence and/or mortality^10^.

Previously, we reported that the three most common diabetes subtypes, namely SAID, MOD, and MARD, cross-sectionally differed in the associations of carbohydrate quality parameters or adherence to dietary patterns with individual cardiometabolic risk factors^11,12^. However, a person’s overall CVD risk can be estimated more accurately using a combined measure considering the weighted effect of multiple CVD risk factors than by only focusing on individual risk traits^13^. Thus, accounting for the competing effects of individual risk predictors within a combined score may improve the identification and risk discrimination of individuals at higher risk of developing CVD^14^.

In the present analyses, we aimed to expand on our previous observations and generate new hypotheses regarding differential associations of i) defined dietary patterns and ii) parameters of carbohydrate quality with the 10-year risk for clinical CVD estimated by the ‘Systematic COronary Risk Evaluation (SCORE)2-Diabetes’ among the diabetes subtypes SAID, MOD, and MARD. SCORE2-Diabetes is an established risk score to estimate the 10-year risk of first occurrence of non-fatal myocardial infarction, stroke, or CVD mortality in an individual with type 2 diabetes in Europe without history of CVD^14^.

## Methods

### Study population

Our analyses were based on data from the German Diabetes Study (GDS), an ongoing observational cohort study^6,15^. As illustrated in Supplementary Figure 1, participants enrolled at the study center in Düsseldorf were eligible for cross-sectional analyses if they had completed the glycemic index-extended food frequency questionnaire, which was implemented in the German Diabetes Study in August 2012. Accordingly, we included participants examined between August 2012 and February 2022 who had a diabetes diagnosis according to the American Diabetes Association criteria^16^ and had participated either in the baseline examination (known diabetes duration <12 months) or the 5-year follow-up examination during this period. For the present cross-sectional analyses, data from both examination time points were combined to maximize statistical power. If data from both examinations were available within this period, only the data from the baseline examination were used. Participants were excluded from analyses if data for variables necessary for allocation to diabetes subtypes were missing (n=37), subtype prevalence was <10% (n=20 SIDD, n=46 SIRD), outcome variables were missing or implausible or prevalent heart attack and/or stroke was documented (n=53), dietary intake data (including energy intake) were implausible (n=5), or potential confounding factors were missing (n=75) (with implausible data comprising clinically impossible values and outliers as identified using graphical methods with Tukey’s approach via boxplots), resulting in a total of 612 participants (n=427 at baseline, n=185 at follow-up) for analyses (Supplementary Figure 1). Importantly, identical examinations at both time points ensured excellent comparability^6,11,12,15^. All participants gave their written informed consent. The study protocol has been approved by the ethics board of the Medical Faculty of Heinrich Heine University (ref. 4508) and the German Diabetes Study is performed according to the Declaration of Helsinki.

### Nutrition assessment

As previously described, dietary intake was assessed using the validated semi-quantitative food frequency questionnaire of the European Prospective Investigation into Cancer and Nutrition (EPIC)-Potsdam study, which assesses habitual food consumption frequencies of 148 food items for an average portion size during the past 12 months^11,12,15,17^. Food group and nutrient intakes were derived from the German Food Code and Nutrient Database (version II.3)^17^.

### Assessment of quality of carbohydrate intake

Using the glycemic index-extended food frequency questionnaire^11,18^, dietary glycemic index and glycemic load were estimated for each participant (for more details see Supplementary Methods). To further characterize carbohydrate intake according to the glycemic index of contributing foods, a cutoff of 55 was used to classify foods as higher glycemic index (≥55, i. e. moderate to high glycemic index) and low glycemic index (<55), based on published glycemic index values^18,19^. Dietary fiber intake was derived solely from the EPIC food frequency questionnaire. In contrast, total sugar (sum of mono- and disaccharides^20^) and total daily energy intake were obtained from the glycemic index-extended food frequency questionnaire, as the version of the EPIC food frequency questionnaire used in the German Diabetes Study does not distinguish between sugar-sweetened and non-sugar-sweetened beverages^11,12,18^.

### Extraction of hypothesis-based dietary patterns

Based on the glycemic index-extended food frequency questionnaire, the traditional Mediterranean diet score, the DASH score, and the plant-based diet indices, i. e. the overall plant-based diet index, the healthful plant-based diet index, and the unhealthful plant-based diet index^21–23^, were generated as previously described^12^ (for more details see Supplementary Methods and Supplementary Table 1).

### Procedures

*Variables necessary for allocation to diabetes subtypes (clustering variables).* The six clustering variables were assessed as described before^6,11,12,15^: Glutamic decarboxylase antibodies were measured by a radioligand assay, hemoglobin A1c (HbA1c) was quantified on a Variant-II (Bio-Rad, Munich, Germany), age at diagnosis was derived from a structured interview, and body mass index was calculated as body weight divided by the square of body height^6,11,12,15^. The homoeostasis model assessment evaluates β-cell function (HOMA2-B) and insulin resistance (HOMA2-IR) using fasting glucose and C-peptide concentrations through the HOMA calculator^24^ (University of Oxford, Oxford, UK).

*Variables necessary for CVD risk estimation.* Using standardized questionnaires, information on current smoking status was assessed^15^. Systolic and diastolic blood pressure were measured three times in the right arm while sitting using a validated automatic device (OMRON 705IT, OMRON HEALTHCARE, Germany). For analyses, the mean of the second and third measurement was taken^25^. Serum concentrations of total and high-density lipoprotein (HDL) cholesterol were measured on a Cobas c311 (Roche Diagnostics, Mannheim, Germany)^15^. Estimated glomerular filtration rate was derived from creatinine and cystatin C^26^.

*Estimation of the absolute 10-year CVD risk.* The 10-year CVD risk was estimated using the low-risk region version of the diabetes-specific SCORE2-Diabetes. This score, an extension of the SCORE2 algorithm, revises the original SCORE model to estimate 10-year CVD risk in Europe^13,14^. SCORE2-Diabetes estimates the 10-year risk of first occurrence of non-fatal myocardial infarction, stroke, or any CVD mortality in an individual with type 2 diabetes in Europe. SCORE2-Diabetes includes variables of the SCORE2 algorithm (age, sex, smoking status, diabetes status, systolic blood pressure, total and HDL cholesterol), complemented by diabetes-specific variables (age at diabetes diagnosis, HbA1c, estimated glomerular filtration rate)^14^. SCORE2-Diabetes was previously applied to a partially overlapping sample within the German Diabetes Study as an exemplary outcome to examine classification uncertainty in type 2 diabetes subtypes^27^.

*Covariates.* Data on age, sex, income, education, and occupation were obtained from standardized questionnaires^15^. The socioeconomic status, which was defined as a multidimensional aggregated score, was derived from information on income, education, and occupation^28^. A physical activity index covering leisure and sport activities as assessed by standardized questionnaires was obtained as a modified version of the Baecke Index^29^. Total daily alcohol intake was assessed from the food frequency questionnaire^17^.

### Statistical analyses

Statistical analyses were conducted using SAS® version 9.4 (SAS Institute, Cary, NC, USA).

*Diabetes subtypes.* As repeatedly applied to the German Diabetes Study^6,11,12,30^, the classification of people with diabetes into one of the five predefined subtypes (clusters) followed the sex-specific rules detailed by Ahlqvist et al.^4^. Briefly, individuals were categorized as SAID, SIDD, SIRD, MOD, or MARD based on six variables, i. e. age and glutamic decarboxylase antibodies at diagnosis, body mass index, HbA1c, HOMA2-B, and HOMA2-IR, using the nearest centroid method. Those who tested positive for glutamic decarboxylase antibodies were allocated to the SAID cluster. Notably, diabetes subtype allocation was made at the respective examination time point^11,12^.

*Comparison of characteristics between diabetes subtypes.* Overall differences in clinical and dietary characteristics between the diabetes subtypes were analyzed using chi-square test for categorical and Kruskal-Wallis test for continuous variables.

*Comparison of absolute estimated CVD risk between diabetes subtypes*. Overall difference in SCORE2-Diabetes was calculated using ANCOVA adjusted for age and sex. As residuals of SCORE2-Diabetes were not linear, SCORE2-Diabetes was entered into the models as ln-transformed variable. Least square means and 95% confidence intervals (CI) were then back-transformed for data presentation.

*Associations of dietary factors with estimated CVD risk and differences between diabetes subtypes.* Multivariable linear regression analyses adjusted for potential confounders was applied to assess associations of dietary factors, i. e. quality of carbohydrate intake, dietary patterns, and the individual food groups constituting each dietary pattern (continuous exposure variable) with SCORE2-Diabetes stratified by diabetes subtype. As residuals of the SCORE2-Diabetes were not linear, SCORE2-Diabetes was entered into the models as ln-transformed variable and effect estimates were then back-transformed for data presentation.

A directed acyclic graph was created using DAGitty v3.1 (https://dagitty.net/) to identify the minimal sufficient set of confounders^31^. With the dietary patterns and the carbohydrate quality parameters as exposure variable and the CVD risk score as outcome variable, the minimal sufficient adjustment set included age, sex, socioeconomic status, physical activity index, and total daily alcohol intake (Suppl. Fig. 2). Thus, model 1 was adjusted for age and sex and model 2 additionally considered socioeconomic status, physical activity index, and total daily alcohol intake (not for models including the Mediterranean diet score). In order to additionally control for total daily energy intake, dietary factors (except for dietary glycemic index) were energy-adjusted using the residual method. Of note, we used the same set of confounders for the analyses with the individual food groups as exposure variable.

Interaction analyses were applied to test for differences between subtypes in associations of dietary factors (parameters of carbohydrate quality, dietary patterns, individual food groups) with SCORE2-Diabetes by adding multiplicative interaction terms (adherence to dietary pattern*diabetes subtypes and parameter of carbohydrate quality*diabetes subtypes, respectively).

*Sensitivity analyses.* To assess reproducibility of the results from association and interaction analyses within a more homogenous cohort, regression analyses were repeated including only individuals from the baseline examination.

## Results

**Fig. 1:**
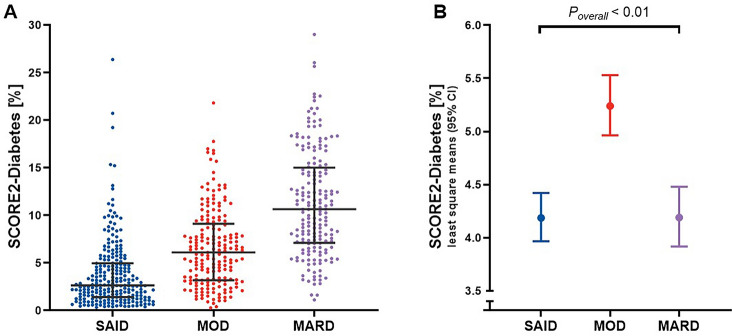
**Crude and adjusted 10-year risk of cardiovascular disease stratified by diabetes subtype**. 10-year risk of cardiovascular disease estimated by SCORE2-Diabetes among people with SAID, MOD, and MARD depicted as (**A**) absolute risk and (**B**) age- and sex-adjusted risk. Data are (**A**) scatter dot plots with median, 25^th^ percentile, 75^th^ percentile and (**B**) least square means with 95% confidence interval. MARD, mild age-related diabetes; MOD, mild obesity-related diabetes; SAID, severe autoimmune diabetes; SCORE, Systematic COronary Risk Evaluation.

*Participants’ characteristics.* A total of 612 individuals with diabetes were included in our cross-sectional study. Of these, 239 (39.1%) were assigned to SAID, 189 (30.9%) to MOD, and 184 (30.1%) to MARD (Table 1). Table 1 presents the clinical characteristics of the three subtypes. Absolute 10-year CVD risk, estimated by SCORE2-Diabetes, was lowest for people with SAID (median (25th percentile; 75th percentile): 2.6% (1.4; 5.0)), intermediate for people with MOD (6.1% (3.2; 9.0)), and highest for people with MARD (10.6% (7.1; 14.9)) (Fig. 1A, Table 1). However, upon age- and sex-adjustment, mean SCORE2-Diabetes was highest for people with MOD and lower (and comparable) for people with SAID and MARD (Fig. 1B).

**Table 1:** Clinical characteristics of the study sample according to diabetes subtype.

	**Total**	**SAID**	**MOD**	**MARD**	***P***_**overall**_
*N (% of study sample)*	*612 (100.0)*	*239 (39.1)*	*189 (30.9)*	*184 (30.1)*	
Age, years	47.5±12.9	39.3±11.7	47.1±9.6	58.7±8.1	<0.01
Age at diabetes diagnosis, years	46.0±12.7	37.8±11.6	45.4±9.5	57.2±7.3	<0.01
Sex (men), n (%)	369 (60.3)	128 (53.7)	100 (52.9)	141 (76.3)	<0.01
Participation time point, n (%)					0.74
Baseline	427 (69.8)	170 (71.1)	128 (67.7)	129 (70.1)	
5-year follow-up	185 (30.2)	69 (28.9)	61 (32.3)	55 (29.9)	
***Cardiovascular risk score***					
SCORE2-Diabetes, %	5.5 (2.6; 9.8)	2.6 (1.4; 5.0)	6.1 (3.2; 9.0)	10.6 (7.1; 14.9)	<0.01
***Cardiovascular risk factors and medication***					
Glucose-lowering medication, n (%)*					<0.01
Insulin (+OAD)	266 (43.5)	209 (87.5)	31 (16.5)	26 (14.1)	
Metformin	165 (27.0)	15 (6.3)	84 (44.7)	66 (35.9)	
Metformin+OAD	17 (2.8)	0 (0.0)	9 (4.8)	8 (4.4)	
Other	28 (4.6)	1 (0.4)	14 (7.5)	13 (7.1)	
None	135 (22.1)	14 (5.9)	50 (26.6)	71 (38.6)	
Antihypertensive medication (yes), n (%)	190 (31.1)	24 (10.0)	86 (45.5)	80 (43.5)	<0.01
Lipid-lowering medication (yes), n (%)	60 (9.8)	10 (4.2)	19 (10.1)	31 (16.9)	<0.01
Hypertension (yes), n (%)	273 (44.6)	66 (27.6)	99 (52.4)	108 (58.7)	<0.01
Diastolic blood pressure, mmHg	83.0±10.1	79.5±9.1	86.7±10.2	83.9±9.8	<0.01
Systolic blood pressure, mmHg	135.9±16.7	128.9±14.3	138.2±15.4	142.5±17.7	<0.01
Hyperlipidemia (yes), n (%)	347 (56.7)	91 (38.1)	136 (72.0)	120 (65.2)	<0.01
Total cholesterol, mg/dL	192 (164; 219)	182 (158; 208)	196 (172; 226)	196 (172; 222)	<0.01
High-density lipoprotein cholesterol, mg/dL	52 (41; 66)	63 (49; 76)	44 (37; 53)	50 (41; 60)	<0.01
Low-density lipoprotein cholesterol, mg/dL	121 (96; 145)	107 (87; 131)	130 (109; 153)	126 (105; 151)	<0.01
Estimated glomerular filtration rate, mL/min per 1.73 m^2^	93.0±15.6	99.8±14.7	90.5±15.9	86.7±13.0	<0.01
***Anthropometric and clinical characteristics***					
Body mass index, kg/m^2^	29.1±6.5	25.6±4.9	35.2±6.1	27.4±3.5	<0.01
Fasting blood glucose, mg/dL	130 (111; 152)	131 (111; 164)	132 (116; 155)	125 (110; 142)	0.02
Fasting blood glucose, mmol/L	7.2 (6.2; 8.4)	7.3 (6.2; 9.1)	7.3 (6.4; 8.6)	6.9 (6.1; 7.9)	0.02
Fasting C-peptide, ng/mL^†^	2 (0.9; 3.1)	0.7 (0.3; 1.2)	3.3 (2.6; 4.2)	2.3 (1.6; 2.9)	<0.01
Fasting C-peptide, nmol/L^†^	0.7 (0.3; 1.0)	0.2 (0.1; 0.4)	1.1 (0.8; 1.4)	0.8 (0.5; 1.0)	<0.01
HOMA2-IR^‡^	1.9 (1.1; 2.8)	0.9 (0.6; 1.4)	2.8 (2.1; 3.5)	1.9 (1.3; 2.4)	<0.01
HOMA2-B^‡^	69.2 (46.6; 97.4)	45.5 (32.1; 65)	86.1 (62.9; 118.5)	72.5 (53.3; 94.8)	<0.01
HbA1c, %	6.4 (5.9; 7.0)	6.6 (6; 7.2)	6.3 (5.9; 7.1)	6.3 (5.9; 6.7)	<0.01
HbA1c, mmol/mol	46.4 (41; 53)	48.6 (42.1; 55.2)	45.3 (41.0; 54.1)	45.3 (41.0; 49.7)	<0.01
Known diabetes duration, months	24.5±28.6	24.1±28.3	25.6±29.4	24±28.4	0.60
Glutamic decarboxylase antibodies positivity at baseline, n (%)	239 (39)	239 (100)	0 (0)	0 (0)	<0.01
***Further covariates***					
Physical activity index (leisure and sport activities)	5.9±1.3	6.2±1.4	5.4±1.1	6.1±1.3	<0.01
Current smoking status (yes), n (%)	125 (20.4)	51 (21.3)	37 (19.6)	37 (20.1)	0.90
Socioeconomic status, n (%)					0.18
Lower	31 (5.1)	15 (6.3)	11 (5.8)	5 (2.7)	
Middle	303 (49.5)	115 (48.1)	102 (54.0)	86 (46.7)	
Upper	278 (45.4)	109 (45.6)	76 (40.2)	93 (50.4)	

Data are presented as n (%), mean ± SD or median (25^th^ percentile; 75^th^ percentile).

Data only available for *n=611 (n=239 SAID, n=188 MOD, n=189 MARD), ^†^n=607 (n=243 SAID, n=189 MOD, n=184 MARD), ^‡^n=511 (n=138 SAID, n=189 MOD, n=184 MARD) individuals, respectively.

*P*_overall_ for differences between the diabetes subtypes using chi-square test for categorical and Kruskal-Wallis test for continuous variables.

HbA1c, hemoglobin A1c; HOMA2-B, homoeostasis model assessment 2 of β-cell function; HOMA2-IR, homoeostasis model assessment 2 of insulin resistance; MARD, mild age-related diabetes; MOD, mild obesity-related diabetes; OAD, oral glucose-lowering drugs; SAID, severe autoimmune diabetes; SCORE, Systematic COronary Risk Evaluation.

Carbohydrate quality was comparable between the three subtypes with a median total carbohydrate intake of 37 energy percentage, split into 16 energy percentage from low glycemic index carbohydrates and 21 energy percentage from higher glycemic index carbohydrates in the total cohort. Adherence to the Mediterranean diet score, the DASH score, and the plant-based diet indices was also similar between the subtypes (Table 2). Table 2 additionally displays the main sources (food groups) of carbohydrate intake and the amounts consumed in each of the food groups constituting the dietary patterns.

**Table 2:** Dietary characteristics of the study sample according to diabetes subtype.

	**Total**	**SAID**	**MOD**	**MARD**	***P***_**overall**_
*N (% of study sample)*	*612 (100.0)*	*239 (39.1)*	*189 (30.9)*	*184 (30.1)*	
***Carbohydrate quality parameters***					
Dietary glycemic index	55.8±3.3	56.0±3.3	56.1±3.1	55.2±3.3	0.02
Glycemic load/1000 kcal	52.7±10.0	52.5±10.3	53.5±8.6	52.2±10.8	0.22
Low glycemic index carbohydrates, gram^a^	77.6 (59.5; 100.5)	79.3 (61.4; 102.9)	75.5 (56.3; 95.7)	76.0 (58.3; 99.7)	0.21
Low glycemic index carbohydrates, energy percentage ^a^	16 (12; 19)	15 (12; 18)	16 (13; 20)	16 (12; 19)	0.55
Higher glycemic index carbohydrates, gram^b^	100.3 (75.9; 149.2)	102.6 (79.6; 163.0)	103.5 (80.7; 139.3)	93.6 (71.0; 138.9)	0.13
Higher glycemic index carbohydrates, energy percentage ^b^	21 (17; 26)	21 (17; 26)	22 (18; 26)	21 (17; 26)	0.36
Dietary fiber, gram	22.5 (17.5; 29.0)	23.4 (17.9; 29.8)	22.1 (17.1; 28.5)	21.7 (17.3; 27.2)	0.18
Dietary fiber/1000 kcal	10.0 (8.5; 11.7)	10.0 (8.7; 11.5)	10.0 (8.4; 11.7)	10.3 (8.7; 12.1)	0.20
Total sugar, gram	83.7 (66.5; 107.3)	86.6 (68.3; 108.9)	83.7 (64.3; 107.2)	81.6 (66.2; 101.6)	0.51
Total sugar, energy percentage	17 (14; 20)	17 (14; 19)	18 (14; 21)	17 (14; 21)	0.06
***Energy and macronutrient intake***					
Total daily energy intake, kcal	1998 (1632; 2482)	2061 (1707; 2606)	1948 (1579; 2437)	1915 (1594; 2401)	0.02
Carbohydrates, energy percentage	38 (34; 41)	38 (34; 41)	38 (35; 41)	37 (34; 42)	0.40
Protein, energy percentage	15 (14; 17)	15 (13; 17)	16 (14; 17)	15 (13; 17)	0.01
Fat, energy percentage	45 (41; 48)	45 (41; 49)	45 (40; 48)	44 (41; 49)	0.61
Alcohol, gram	6.0 (2.0; 16.2)	8.6 (3.0; 17.0)	3.4 (1.2; 11.0)	7.4 (2.2; 21.0)	<0.01
***Main sources of carbohydrate intake***					
Cereal (products), energy percentage	13.7 (10.9; 18.1)	14.2 (11.1; 19.0)	14.0 (11.2; 17.7)	12.6 (10.3; 17.3)	0.04
Fruit, energy percentage	4.4 (2.8; 6.9)	4.4 (2.8; 6.8)	4.3 (2.8; 6.5)	4.5 (2.5; 7.4)	0.68
Sugar and confectionary, energy percentage	3.4 (2.3; 5.0)	3.5 (2.5; 4.9)	3.6 (2.4; 5.1)	3.1 (1.9; 4.9)	0.09
Dairy products, energy percentage	2.7 (1.8; 4.0)	2.6 (1.7; 3.9)	2.6 (1.8; 4.2)	2.8 (1.8; 4.0)	0.73
Cake, energy percentage	2.5 (1.7; 3.8)	2.5 (1.7; 3.8)	2.6 (1.7; 4.3)	2.4 (1.5; 3.6)	0.20
***Dietary patterns***					
Mediterranean diet score	4 (3; 5)	4 (3; 6)	4 (3; 5)	4 (3; 6)	0.15
DASH score	24 (21; 27)	24 (21; 27)	23 (20; 27)	24 (21; 28)	0.14
Overall plant-based diet index	54 (49; 58)	54 (49; 58)	53 (49; 57)	54 (51; 59)	0.10
Healthful plant-based diet index	53 (48.5; 59)	54 (49; 59)	52 (47; 57)	55 (49; 60)	<0.01
Unhealthful plant-based diet index	54 (48; 59)	53 (47; 59)	54 (48; 60)	54 (48; 59)	0.31
***Food groups contributing to the Mediterranean diet score***					
Fruit and nuts, gram	175 (105; 264)	186 (104; 263)	168 (104; 289)	175 (111; 252)	0.86
Vegetables, gram	215 (164; 287)	219 (167; 298)	217 (168; 300)	209 (159; 265)	0.21
Legumes, gram	4.1 (3.5; 8.7)	3.9 (3.5; 8.6)	4.1 (3.5; 8.7)	6.1 (3.6; 8.7)	0.02
Cereals, gram	160 (121; 261)	176 (129; 279)	159 (125; 236)	141 (108; 248)	<0.01
Fish, gram	17 (6; 33)	15 (6; 26)	17 (6; 33)	26 (6; 39)	0.05
Dairy products, gram*	225 (141; 362)	232 (144; 382)	206 (144; 347)	224 (130; 318)	0.46
Meat, gram	133 (87; 197)	134 (81; 195)	140 (96; 222)	126 (86; 176)	0.05
Monounsaturated-to-polyunsaturated fat ratio, arbitrary units	0.93 (0.86; 1.03)	0.93 (0.85; 1.04)	0.93 (0.86; 1.00)	0.96 (0.87; 1.05)	0.16
Alcohol from alcoholic beverages, gram	5.3 (1.3; 15.2)	7.8 (2.1; 16.3)	2.8 (0.6; 10.2)	6.3 (1.3; 20.4)	<0.01
***Food groups contributing to the DASH score***					
Fruit, gram	167 (99; 259)	173 (94; 257)	161 (99; 281)	162 (102; 243)	0.98
Vegetables, gram	215 (164; 287)	219 (167; 298)	217 (168; 300)	209 (159; 265)	0.21
Nuts and legumes, gram	10.5 (5.5; 19.5)	10.8 (5.7; 20.3)	9.5 (4.9; 15.5)	11.1 (6.5; 24.6)	<0.01
Whole grain bread, gram*	67 (43; 136)	68 (41; 154)	67 (43; 128)	68 (43; 118)	0.79
Low-fat dairy, gram	21 (0; 80)	19 (0; 79)	26 (0; 80)	21 (0; 82)	0.33
Red meat, gram	61 (29; 104)	58 (26; 104)	71 (32; 122)	60 (36; 92)	0.22
Sugar-sweetened beverages, gram	5.5 (0; 14.3)	5.5 (0; 14.3)	5.5 (0.0; 14.3)	5.5 (5.5; 14.3)	<0.01
Sodium, gram	2.4 (1.9; 3.1)	2.5 (1.9; 3.1)	2.5 (2.0; 3.1)	2.3 (1.9; 2.9)	0.13
***Food groups contributing to the plant-based diet indices***					
*Healthful plant-based foods*					
Whole grain bread, gram*	68 (43; 136)	69 (43; 156)	67 (43; 128)	68 (50; 131)	0.62
Fruit, gram	167 (99; 259)	173 (94; 257)	161 (99; 281)	162 (102; 243)	0.98
Vegetables, gram	215 (164; 287)	219 (167; 298)	217 (168; 300)	209 (159; 265)	0.21
Nuts, gram	3.0 (1.0; 8.2)	4.9 (1.1; 15.2)	2.9 (0.9; 6.8)	3.4 (1.0; 15.6)	<0.01
Legumes, gram	4.1 (3.5; 8.7)	3.9 (3.5; 8.6)	4.1 (3.5; 8.7)	6.1 (3.6; 8.7)	0.02
Vegetable oils, gram	13 (9; 19)	14 (10; 20)	13 (8; 19)	12 (9; 18)	0.07
Tea and coffee, gram	764 (397; 1113)	754 (355; 1114)	665 (389; 1031)	846 (437; 1175)	0.01
*Less healthful plant-based foods*					
Fruit juices, gram	16 (3; 54)	16 (3; 53)	20 (7; 60)	10 (3; 41)	0.02
Refined grains, gram	50 (33; 86)	55 (38; 94)	57 (40; 89)	42 (22; 57)	<0.01
Potatoes, gram	50 (28; 95)	49 (33; 93)	48 (26; 94)	56 (31; 102)	0.14
Sugar-sweetened beverages, gram	5.5 (0.0; 14.3)	5.5 (0.0; 14.3)	5.5 (0.0; 14.3)	5.5 (5.5; 14.3)	<0.01
Sweets and desserts, gram	59 (42; 91)	64 (45; 95)	60 (42; 93)	53 (41; 84)	0.04
*Animal-based foods*					
Animal fat, gram	15 (5; 25)	16 (6; 25)	13 (5; 24)	14 (4; 24)	0.05
Dairy products, gram*	195 (114; 336)	204 (115; 354)	185 (115; 330)	194 (112; 306)	0.56
Egg, gram	18 (9; 21)	19 (10; 21)	19 (10; 21)	18 (8; 20)	0.06
Fish or seafood, gram	20 (7; 44)	20 (7; 32)	20 (7; 44)	32 (7; 45)	0.06
Meat, gram	133 (87; 197)	134 (81; 195)	140 (96; 222)	126 (86; 176)	0.05
Misc. animal-based foods, gram	5.5 (2.2; 8.8)	5.8 (3.0; 9.5)	5.9 (2.2; 9.1)	4.5 (2.1; 6.6)	<0.01

Data are presented as n (%), mean ± SD or median (25^th^ percentile; 75^th^ percentile).

^a^ Low glycemic index food sources are defined as ≤55.

^b^ Higher glycemic index food sources are defined as >55.

* See Supplementary Table 1 for differences in food group definitions across dietary patterns.

*P*_overall_ for differences between the diabetes subtypes using chi-square test for categorical and Kruskal-Wallis test for continuous variables.

DASH, Dietary Approaches to Stop Hypertension; MARD, mild age-related diabetes; MOD, mild obesity-related diabetes; SAID, severe autoimmune diabetes.

**Fig. 2:**
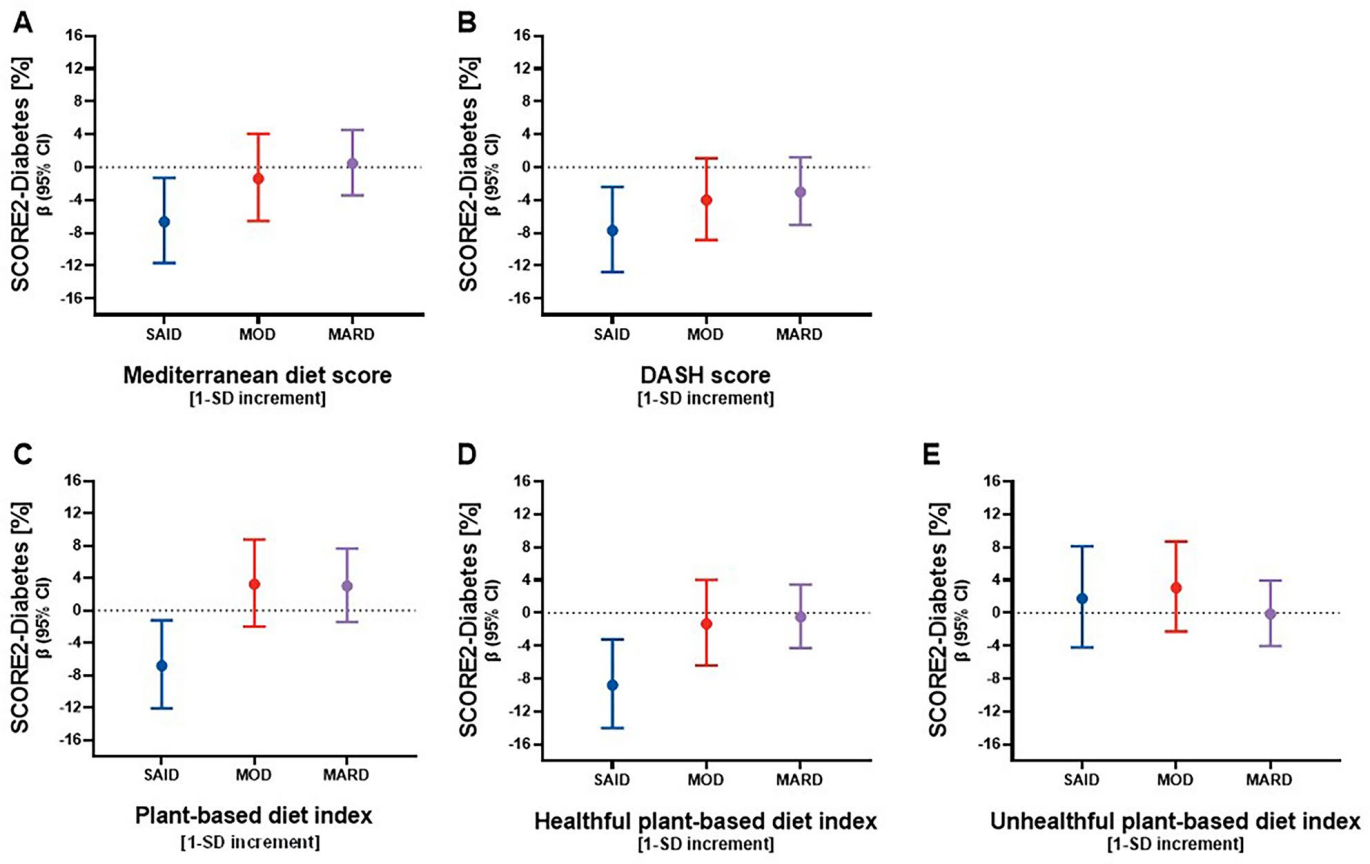
**Associations of dietary patterns with the estimated 10-year cardiovascular risk stratified by diabetes subtype**. Regression coefficients with 95% CI for associations of (**A**) Mediterranean diet score, (**B**) DASH score, (**C**) overall plant-based diet index, (**D**) healthful plant-based diet index, and (**E**) unhealthful plant-based diet index with SCORE2-Diabetes. Adjusted for age, sex, socioeconomic status, physical activity index, and total daily alcohol intake (not for models including the Mediterranean diet score as exposure variable). Dietary patterns were energy adjusted using the residual method. Regression coefficients should be interpreted as follows: relative change of SCORE2-Diabetes per increment of 1 standard deviation in dietary pattern score (Example: An increment of 1 standard deviation in adherence to the Mediterranean diet score (energy adjusted), i. e. an increase by 1.61, is associated with a relative decrease in SCORE2-Diabetes by -6.6% (-11.7; -1.3) among SAID). 1 standard deviation of the Mediterranean diet score (energy adjusted) = 1.61; 1 standard deviation of the DASH score (energy adjusted) = 4.79; 1 standard deviation of the overall plant-based diet index (energy adjusted) = 6.39; 1 standard deviation of the healthful plant-based diet index (energy adjusted) = 7.19; 1 standard deviation of the unhealthful plant-based diet index (energy adjusted) = 7.54. Data are available for n=612 (n=239 SAID, n=189 MOD, n=184 MARD) individuals. Exact values for all comparisons are provided in Supplementary Table 2. CI, confidence interval; DASH, Dietary Approaches to Stop Hypertension; MARD, mild age-related diabetes; MOD, mild obesity-related diabetes; SAID, severe autoimmune diabetes; SCORE, Systematic COronary Risk Evaluation; SD, standard deviation.

*Associations of dietary factors with estimated CVD risk stratified by diabetes subtypes.* Regarding the dietary patterns, among people with SAID, an increment of 1 standard deviation in adherence to the Mediterranean diet score, the DASH score, the overall plant-based diet index, and the healthful plant-based diet index was associated with a relative decrease in SCORE2-Diabetes by -6.6% (95% CI: -11.7; -1.3), -7.7% (95% CI: -12.7; -2.4), -6.8% (95% CI: -12.1; -1.2), and -8.8% (95% CI: -14.0; -3.2), respectively, translating into a low absolute CVD risk decrease. No clear association was evident for the unhealthful plant-based diet index (Fig. 2, Supplementary Table 2). The associations of the overall plant-based diet index and the healthful plant-based diet index with SCORE2-Diabetes were subtype-specific (*P*_interaction_<0.01 and *P*_interaction_=0.04, respectively) (Supplementary Table 2). Among people with MOD and MARD, no statistically significant associations were observed for any of the dietary patterns with SCORE2-Diabetes. However, a higher adherence to the DASH score pointed to lower and a higher adherence to the overall plant-based diet index pointed to higher SCORE2-Diabetes, but 95% CIs included the null value (Fig. 2, Supplementary Table 2).

On the food group level, among people with SAID, higher intake of ‘nuts’, legumes’, and ‘nuts and legumes’ was associated with lower SCORE2-Diabetes (ß (95% CI) per increment of 1 standard deviation: -5.9% (95% CI: -10.6; -0.9), -6.4% (95% CI: -11.5; -1.1), -7.7% (95% CI: -12.4; -2.7), respectively). Also, each increment of 1 standard deviation in intake of ‘egg’ and ‘miscellaneous animal-based foods’ was associated with a relative increase in SCORE2-Diabetes by 6.6% (95% CI: 1.2; 12.2) and 5.6% (95% CI: 0.0; 11.4), respectively. Among people with MOD, each increment of 1 standard deviation in ‘tea and coffee’ was associated with a relative increase in SCORE2-Diabetes by 9.6% (95% CI: 4.8; 14.5) and among people with MARD, each increment of 1 standard deviation in ‘tea and coffee’ and ‘meat’ was associated with a relative increase in SCORE2-Diabetes by 4.8% (95% CI: 0.4; 9.3) and 5.0% (95% CI: 0.2; 10.1), respectively (Supplementary Table 3).

**Fig. 3:**
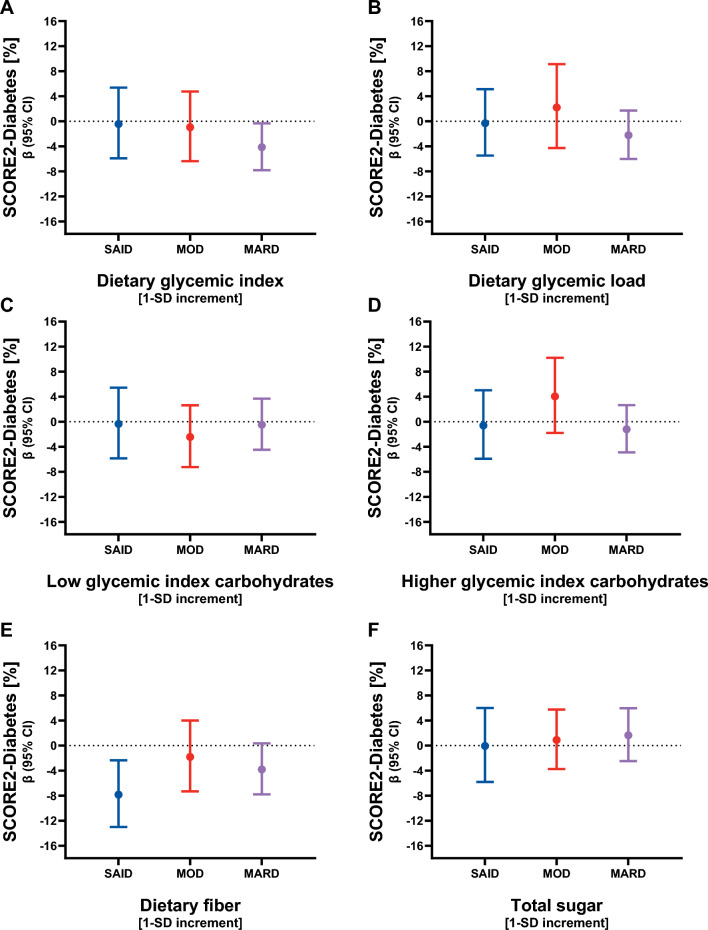
**Associations of carbohydrate quality parameters with the estimated 10-year cardiovascular risk stratified by diabetes subtype**. Regression coefficients with 95% CI for associations of (**A**) dietary glycemic index, (**B**) dietary glycemic load, (**C**) low glycemic index carbohydrates, (**D**) higher glycemic index carbohydrates, (**E**) dietary fiber, and (**F**) total sugar with SCORE2-Diabetes. Adjusted for age, sex, socioeconomic status, physical activity index, and total daily alcohol intake. Carbohydrate quality parameters (except dietary GI) were energy adjusted using the residual method. (**C**) Low glycemic index food sources are defined as ≤55. (**D**) Higher glycemic index food sources are defined as >55. Regression coefficients should be interpreted as follows: relative change of SCORE2-Diabetes per 1-standard deviation increment in carbohydrate quality parameter (Example: An increment of 1 standard deviation in dietary fiber (energy adjusted), i. e. an increase by 6.35 g, is associated with a relative decrease in SCORE2-Diabetes by -7.1% (-13.4; -0.4) among SAID). 1 standard deviation of dietary glycemic index = 3.26; 1 standard deviation of dietary glycemic load (energy adjusted) = 21.78; 1 standard deviation of low glycemic index carbohydrates (energy adjusted) = 27.15 g; 1 standard deviation of higher glycemic index carbohydrates (energy adjusted) = 36.96 g; 1 standard deviation of dietary fiber (energy adjusted) = 6.35 g; 1 standard deviation of total sugar (energy adjusted) = 25.23 g. Data are available for n=612 (n=239 SAID, n=189 MOD, n=184 MARD) individuals. Exact values for all comparisons are provided in Supplementary Table 4. CI, confidence interval; MARD, mild age-related diabetes; MOD, mild obesity-related diabetes; SAID, severe autoimmune diabetes; SCORE, Systematic COronary Risk Evaluation; SD, standard deviation.

For the carbohydrate quality parameters, no statistically significant associations were observed between dietary glycemic load, low glycemic index carbohydrates, higher glycemic index carbohydrates, and total sugar with SCORE2-Diabetes in any subtype. Among people with SAID, an increment of 1 standard deviation in dietary fiber was associated with a relative decrease in SCORE2-Diabetes by -7.8% (95% CI: -13.0; -2.4), which again translates into a low absolute 10-year CVD risk decrease, while this association was not evident in MOD. Among MARD, a higher dietary fiber intake pointed to a lower SCORE2-Diabetes, but 95% CIs included the null value. The *P*-value for the interaction between diabetes subtype and dietary fiber was 0.07. Also, among MARD, a higher dietary glycemic index was associated with lower SCORE2-Diabetes (ß (95% CI) per increment of 1 standard deviation: -4.1% (-7.8; -0.3)), while this association was not evident in SAID and MOD (Fig. 3, Supplementary Table 4).

*Sensitivity analysis.* Comparing the main results with those of the sub-cohort comprising only individuals at baseline, associations were largely comparable based on their effect estimates and 95% CIs as shown, for example, by the association of higher Mediterranean diet score with lower SCORE2-Diabetes among SAID (ß (95% CI)_main analysis_: -6.6% (-11.7; -1.3), ß (95% CI)_sensitivity analysis_: -5.3% (-11.4; 1.3), respectively) or the association of higher dietary fiber with lower SCORE2-Diabetes among SAID (ß (95% CI)_main analysis_: -7.8% (-13.0; -2.4), ß (95% CI)_sensitivity analysis_: -7.2% (-13.5; -0.4), respectively). Among MOD, however, effect estimates for the association of higher overall plant-based diet index with SCORE2-Diabetes differed between the main cohort and the sub-cohort (ß (95% CI)_main analysis_: 3.3% (-2.0; 8.8), ß (95% CI)_sensitivity analysis_: 7.1% (0.6; 14.0), respectively) (Supplementary Tables 5,6).

## Discussion

The main observations of the present cross-sectional analyses generating new hypotheses regarding the differential associations of dietary factors with the 10-year CVD risk, estimated by SCORE2-Diabetes, in the diabetes subtypes SAID, MOD, and MARD were as follows: (i) In people with SAID, higher adherence to healthy dietary patterns and a higher dietary fiber intake were associated with lower future CVD risk. However, the absolute risk reduction was small, likely reflecting the already low estimated 10-year CVD risk in this subtype. (ii) In people with MOD and MARD, associations between the dietary factors analyzed and the future CVD risk were inconclusive. (iii) Associations of the overall plant-based diet index and the healthful plant-based diet index with SCORE2-Diabetes differed across the three subtypes.

Of the three subtypes included, people with MARD had the highest estimated absolute 10-year CVD risk. This is in line with prospective observations of highest risk for coronary events and stroke in people with MARD compared to those with SAID and MOD^4^. In addition, in people with type 2 diabetes and prevalent CVD, the risk for a composite of nonfatal stroke, nonfatal myocardial infarction, and cardiovascular death (3-point major adverse cardiovascular events) was higher in MARD than in MOD^32^. However, the increased CVD risk among people with MARD was no longer apparent after age- and sex-adjustment in our cohort, which is consistent with previous longitudinal observations^4^. Of note, we observed most associations between dietary factors for SAID, the subtype with the lowest 10-year CVD risk. In this subtype, higher adherence to healthy dietary patterns and higher dietary fiber intake were linked to lower estimated 10-year CVD risk. Of the individual food groups constituting the dietary patterns, nuts and legumes – which provide dietary fiber as well as multiple other cardioprotective bioactive components – have been associated with improved cardiometabolic risk factors and decreased CVD risk^33^ and appeared to contribute most to the observed inverse association of the dietary patterns with SCORE2-Diabetes.

These observations expand our previous reports in which we investigated associations with individual CVD risk factors that are used to calculate SCORE2-Diabetes^14^. Briefly, in people with SAID, higher adherence to healthy dietary patterns and higher intake of dietary fiber were associated with lower systolic blood pressure and an improved serum lipid profile, while we observed fewer associations for MOD and MARD^11,12^. Importantly, additional associations were evident for these two subtypes with e. g. high-sensitivity C-reactive protein and the fatty liver index^11,12^, but these variables are not part of SCORE2-Diabetes^14^. Nevertheless, concentrations of high-sensitivity C-reactive protein are predictive of incident cardiovascular events^34^ and the hepatic lipid content associates with cardiovascular risk^35^.

In people with MOD from the baseline examination only, higher adherence to the overall plant-based diet index was associated with higher SCORE2-Diabetes. Thus, higher intake of plant-based foods – if not differentiating between healthy (e. g. whole grains, fruits, vegetables, legumes^22^) and less healthy sources (e. g. refined grains, fruit juices, sugar-sweetened beverages, sweets and desserts^22^) – might not be favorably associated with the estimated 10-year CVD risk in this subtype.

Since the clinically implausible inverse association of the dietary glycemic index and SCORE2-Diabetes among MARD was confined to the fully-adjusted model of the main analysis, but not confirmed in its age- and sex-adjusted model and attenuated in the analyses of the baseline cohort, we assume that this represents a chance finding.

Reasons for the null associations of the dietary patterns and the remaining carbohydrate quality parameters among individuals with MOD and MARD need to be elucidated in further studies with larger sample sizes. The precision nutrition approach requires refinement of dietary recommendations specifically for those (groups of) individuals who benefit less from standard healthful dietary recommendations^36^; because optimizing dietary patterns through personalized care enhances the management of obesity and diabetes^37^. A different personalized nutrition approach, however suggests that better health outcomes are achieved by supporting people more effectively in achieving their individual goals by providing in situ and just-in-time advice rather than by perfectly personalized dietary recommendations^38^.

Overall, the present observations in people with SAID suggesting that adherence to healthy dietary patterns such as the Mediterranean, DASH, or a healthful plant-based diet as well as a high dietary fiber intake are associated with lower future CVD risk, align with the current dietary recommendations for people with diabetes^39–42^. Additional research is needed not only to determine whether subtype-specific dietary recommendations as part of personalized nutritional concepts (complementing population-based advice) can improve diabetes management and reduce complications^36^, but also to develop approaches how people can be empowered to achieve their individual goals^38^.

### Strengths and limitations

The strengths of our study include the in-depth phenotyping and the consideration of different dietary aspects, namely the derivation of dietary patterns that account for the synergistic effects of individual foods and nutrients and the precise carbohydrate intake assessment, covering both carbohydrate quantity and quality. The methodological strengths of the assessment of the glycemic index and glycemic load in the German Diabetes Study have been previously described^11^. A limitation of our study is that we did not assess actual CVD incidence but instead used a validated CVD risk prediction score as a proxy^14^. While SCORE2-Diabetes has demonstrated good agreement with the expected 10-year CVD incidence^14^, it may not fully capture individual future CVD outcomes. Nevertheless, as stated above, our cross-sectional observations of the highest absolute estimated 10-year CVD risk in people with MARD corresponds to previous prospective observations of highest CVD incidence in this subtype^4,32^. Of course, no causality can be inferred from our data due to the cross-sectional study design and the present observations, which were derived based on the hypothesis-generating approach of cross-sectional associations using an estimated CVD risk score as the outcome measure, need to be confirmed in longitudinal studies using validated incident CVD events as endpoints. Further limitations of our study regarding dietary intake assessment are that these data are self-reported by food frequency questionnaire, thus potentially prone to misreporting^43^. Food frequency questionnaires are especially susceptible to recall bias due to their reliance on long-term memory on the habitual food consumption frequencies of the last e. g. 12 months. However, there is no indication of differential bias in our cohort. Nevertheless, we acknowledge that individuals with recently diagnosed diabetes might have changed their diet recently and that misreporting of socially desirable foods – those classified as healthy during dietary counselling – might have occurred^44^. Regarding the glycemic index values, since their availability from European foods is still limited, data must be supplemented with values from Australian and American food items^18,19^. Also, the food frequency questionnaire does not assess factors considerably influencing the glycemic index of a food (e. g. variety, cooking methods, ripeness)^18^. Although we adjusted for several confounders using a directed acyclic graph-based approach, some residual confounding may remain. Moreover, measurement error inherent to the food frequency questionnaire and the glycemic index assessment could have led to exposure misclassification and, consequently, attenuation or distortion of the observed associations. Diabetes subtype distribution might have been affected by the distinct inclusion and exclusion criteria of the German Diabetes Study, which may partly explain the low absolute number of people with SIDD and SIRD, which we excluded as they could not be considered for meaningful analyses. The HbA1c cut-off of 9.0%, for instance, might have led to exclusion of a number of persons belonging to SIDD^11,15,45^. However, this study design of the German Diabetes Study, unlike population-based designs, results in a rather large number of individuals with autoimmune diabetes. Still, subtype distribution within the German Diabetes Study, specifically for MOD and MARD, aligns with cohorts with population-based recruitment^46^. Although other concepts for reclassification of diabetes^47^ might lead to different results, both phenotypic and genotypic clustering approaches yielded clusters with similar properties^5^. Finally, as participants of the German Diabetes Study are predominantly of European ancestry, the generalizability of our results to populations with different ethnic backgrounds might be limited.

## Conclusions

In conclusion, in people with SAID, higher adherence to healthy dietary patterns and a higher intake of dietary fiber were associated with lower estimated 10-year CVD risk, while in MOD and MARD, no clear associations were observed between the dietary factors analyzed and the future CVD risk. Due to the hypothesis-generating approach of this study and its cross-sectional design, longitudinal analyses with larger samples are warranted to confirm these observations and to evaluate whether dietary factors are differentially associated with future CVD risk in the subtypes. Additionally, intervention studies are needed to explore how people can be effectively supported in implementing dietary changes to achieve their individual health outcomes.

## Supplementary Information

Supplementary Information.

## Data Availability

Due to restrictions imposed by the ethics committee of Heinrich Heine University Düsseldorf regarding patient consent, data are available upon request. Requests for data may be sent to Prof. Dr. Michael Roden (Michael.Roden@ddz.de), PI of the GDS.

## Abbreviations

CI: Confidence interval
CVD: Cardiovascular disease
DASH: Dietary approaches to stop hypertension
EPIC: European prospective investigation into cancer and nutrition
HbA1c: Hemoglobin A1c
HDL: High-density lipoprotein
HOMA2-B: Homoeostasis model assessment of β-cell function
HOMA2-IR: Homoeostasis model assessment of insulin resistance
MARD: Mild age-related diabetes
MOD: Mild obesity-related diabetes
SAID: Severe autoimmune diabetes
SCORE: Systematic COronary Risk Evaluation
SIDD: Severe insulin-deficient diabetes
SIRD: Severe insulin-resistant diabetes
