# Supplementary material for: Differential associations of dietary patterns with estimated 10-year cardiovascular risk in diabetes subtypes
Source: Sci Rep. 2026 Jul 6;16:20783. doi: 10.1038/s41598-026-57026-y (PMC13338200; doi:10.1038/s41598-026-57026-y)
Supplement: Supplementary file 1 — Supplementary Information. [file 41598_2026_57026_MOESM1_ESM.docx]

**SUPPLEMENTARY MATERIAL**

**Differential associations of dietary patterns with estimated 10-year cardiovascular risk in diabetes subtypes**

Katharina S. Weber^1^, Sabrina Schlesinger^2,3^, Alexander Lang^2,3^, Janina Goletzke^5^, Cara Övermöhle^1^, Eike A. Strathmann^1^, Klaus Straßburger^2,3^, Nitika Singh^3,4^, Oana-Patricia Zaharia^3,4,6^, Sandra Trenkamp^3,4^, Robert Wagner^3,4,6^, Wolfgang Lieb^1^, Anette E. Buyken^5^, Michael Roden^6,4,3^, Christian Herder^3,4,6*^, for the GDS group^†^

^1^Institute of Epidemiology, Kiel University, Kiel, Germany; ^2^Institute for Biometrics and Epidemiology, German Diabetes Center, Leibniz Center for Diabetes Research at Heinrich Heine University Düsseldorf, Düsseldorf, Germany; ^3^German Center for Diabetes Research (DZD), Partner Düsseldorf, München-Neuherberg, Germany; ^4^Institute for Clinical Diabetology, German Diabetes Center, Leibniz Center for Diabetes Research at Heinrich Heine University Düsseldorf, Düsseldorf, Germany; ^5^Faculty of Natural Sciences, Institute of Nutrition, Consumption and Health, Paderborn University, Paderborn, Germany; ^6^Department of Endocrinology and Diabetology, Medical Faculty and University Hospital Düsseldorf, Heinrich Heine University Düsseldorf, Düsseldorf, Germany.

*Corresponding author:

Dr. Katharina Weber

Institute of Epidemiology, Kiel University

Niemannsweg 11

D 24105 Kiel, Germany

Katharina.Weber@epi.uni-kiel.de

p: +49 431 500 30237, f: +49 431 500 30204

**SUPPLEMENTARY METHODS**

***Assessment of quality of carbohydrate intake.*** In order to more accurately estimate the dietary glycemic index in individuals with diabetes, the food frequency questionnaire of the EPIC study was extended by questions specific for the glycemic index [1, 2]. This glycemic index extended food frequency questionnaire has been developed and validated for individuals with diabetes within the German Diabetes Study (GDS) [2]. Briefly, carbohydrate-rich food groups with substantial variations in the glycemic index of their individual foods were identified from the food frequency questionnaire, including breakfast cereals, bread and buns, pasta and rice, potatoes and potato products, juices and lemonades, savory snacks, and cakes. The glycemic index extended questionnaire listed the most frequently consumed items by German adults, using the same consumption frequency categories as the food frequency questionnaire. Published glycemic index values were assigned to the carbohydrate-containing food items of both, the glycemic index extended questionnaire and the food frequency questionnaire itself.

***Extraction of hypothesis-based dietary patterns.*** The traditional Mediterranean diet score, the DASH score, and the plant-based diet indices [3–5] were generated as previously described [6] and as shown in more detail in Supplementary Table 1. Briefly, the Mediterranean diet score ranges from 0 (minimal adherence) to 9 (maximal adherence) and assesses adherence based on the consumption of key food groups such as vegetables, legumes, fruit, cereals, fish, low-to-moderate alcohol consumption, while reducing meat and dairy products, and additionally considering the lipid ratio (monounsaturated to saturated fatty acids) [3] (Supplementary Table 1). The DASH score ranges from 8 to 40 and emphasizes the intake of fruit, vegetables, nuts and legumes, whole grains (reflected by whole grain bread), low-fat dairy, while limiting sodium, sugar-sweetened beverages, and red meat [5] (Supplementary Table 1). The overall plant-based diet index, the healthful plant-based diet index, and the unhealthful plant-based diet index each range from 18 to 90 and evaluate the consumption of plant-based vs. animal-based food groups (animal fat, dairy, egg, fish or seafood, meat, miscellaneous animal-based foods) by distinguishing between healthful plant food groups (whole grains, fruit, vegetables, nuts, legumes, vegetable oils, tea and coffee) and less healthful plant food groups (fruit juices, refined grains, potatoes, sugar-sweetened beverages, sweets and desserts) [4] (Supplementary Table 1).

**SUPPLEMENTARY FIGURE LEGENDS**

**Supplementary Figure 1:** Flowchart

Flowchart depicting the selection of individuals eligible for analysis.

FU, follow-up.

**Supplementary Figure 2:** Directed acyclic graph for the association of dietary factors (parameters of carbohydrate quality and dietary patterns) with the estimated 10-year cardiovascular risk.

BMI, body mass index.

**SUPPLEMENTARY FIGURES**

**Supplementary Figure 1:** Flowchart depicting the selection of individuals eligible for analysis

**Supplementary Figure 2:** Directed acyclic graph for the association of dietary factors (parameters of carbohydrate quality and dietary patterns) with the estimated 10-year cardiovascular risk

**SUPPLEMENTARY TABLES**

**Supplementary Table 1:** Food items constituting the food groups of the dietary patterns

| **Food groups** | **Food items of the respective food group** |
| --- | --- |
| ***Mediterranean diet score*** |  |
| Fruit and nuts | Fruits, nuts and seeds |
| Vegetables | Leafy vegetables, fruiting vegetables, root vegetables, cabbages, mushrooms, grain and pod vegetables, onion and garlic, stalk vegetables and sprouts, mixed salad and mixed vegetables |
| Legumes | Legumes |
| Cereals | Flour, flakes, starches, and semolina, pasta, rice, and other grain, bread, crispbread and rusks, breakfast cereals, salty biscuits and aperitif biscuits, dough and pastry |
| Fish | Fish, crustaceans and mollusks |
| Dairy products | Milk, milk beverages, yoghurt, fromage blank and petits suisses, cheeses, cream desserts and puddings, dairy creams, milk for coffee and creamers |
| Meat | Beef, pork, mutton and lamb, game, offals, poultry, processed meat |
| Saturated fatty acids | Saturated fatty acids |
| Monounsaturated fatty acids | Monounsaturated fatty acids |
| Alcohol | Alcohol from alcoholic beverages (i. e. wine, fortified wines, beer and cider, spirits and brandy, aniseed drinks, liquors, cocktails and punches) |
|  |  |
| ***Dietary Approaches to Stop Hypertension (DASH) score*** |  |
| Fruit | Fruits, mixed fruits |
| Vegetables | Leafy vegetables, fruiting vegetables, root vegetables, cabbages, mushrooms, grain and pod vegetables, onion and garlic, stalk vegetables and sprouts, mixed salad and mixed vegetables |
| Nuts and legumes | Nuts and seeds, legumes, soya products |
| Whole grain bread | Whole grain bread, gray rye bread, crispbread (food group score was set to 0 if whole grain bread intake was 0 g/d, giving particular weight to this food item) |
| Low-fat dairy | Low-fat milk, low-fat cocoa, low-fat yoghurt, buttermilk, kefir, low-fat fruit yoghurt, low-fat soft cheese, low-fat hard cheese |
| Red meat | Beef, pork, mutton and lamb, game, offals |
| Sugar-sweetened beverages | Sugar-sweetened carbonated/ soft/ isotonic drinks |
| Sodium | Sodium |
|  |  |
| ***Plant-based diet indices*** |  |
| *Healthful plant-based foods* |  |
| Whole grain bread | Whole grain bread, gray rye bread, crispbread |
| Fruit | Fruits, mixed fruits |
| Vegetables | Leafy vegetables, fruiting vegetables, root vegetables, cabbages, mushrooms, grain and pod vegetables, onion and garlic, stalk vegetables and sprouts, mixed salad and mixed vegetables |
| Nuts | Nuts and seeds |
| Legumes | Legumes, soya products |
| Vegetable oils | Vegetable oils |
| Tea and coffee | Coffee, tea, herbal tea, chicory and coffee substitutes |
| *Less healthful plant-based foods* |  |
| Fruit juices | Fruit and vegetable juices |
| Refined grains | Pasta, rice, and, other grain, white bread, bread rolls, and toast, pancakes |
| Potatoes | Potatoes |
| Sugar-sweetened beverages | Sugar-sweetened carbonated/ soft/ isotonic drinks |
| Sweets and desserts | Sugar, honey, and jam, chocolate, candy bars, and paste, confectionary non chocolate, syrup, water ice and sorbet, cakes, pies, pastries, and puddings, dry cakes and biscuits |
| *Animal-based foods* |  |
| Animal fat | Butter, marine oil, other animal fat |
| Dairy products | Milk, milk beverages, yoghurt, fromage blank and petits suisses, cream desserts and puddings, dairy creams, milk for coffee and creamers, ice cream |
| Egg | Egg |
| Fish or seafood | Fish, crustaceans and mollusks, fish products and fish in crumbs |
| Meat | Red meat, poultry, game, processed meat, offals |
| Miscellaneous animal-based foods | Casseroles, baguettes and pizza, mayonnaise and similar products |

**Supplementary Table 2:** Associations of dietary patterns with the 10-year cardiovascular risk estimated by the Systematic COronary Risk Evaluation (SCORE)2-Diabetes stratified by diabetes subtype

| **SCORE2-Diabetes** | **SAID**  **(n=239)** | | **MOD**  **(n=189)** | | **MARD**  **(n=184)** | | *P*_int_* |
| --- | --- | --- | --- | --- | --- | --- | --- |
|  | ß (95% CI) | *P* | ß (95% CI) | *P* | ß (95% CI) | *P* |  |
| **Mediterranean diet score** (energy adjusted) |  |  |  |  |  |  |  |
| *Model 1* | -8.2 (-13.1; -3.1) | <0.01 | -4.2 (-9.2; 1.0) | 0.11 | -2.8 (-6.8; 1.3) | 0.18 |  |
| *Model 2* | -6.6 (-11.7; -1.3) | 0.02 | -1.4 (-6.6; 4.1) | 0.61 | 0.5 (-3.4; 4.5) | 0.82 | 0.21 |
| **DASH score** (energy adjusted) |  |  |  |  |  |  |  |
| *Model 1* | -9.2 (-14.0; -4.2) | <0.01 | -6.2 (-10.8; -1.3) | 0.01 | -6.3 (-10.3; -2.2) | <0.01 |  |
| *Model 2* | -7.7 (-12.7; -2.4) | 0.01 | -4.0 (-8.8; 1.1) | 0.12 | -3.0 (-7.0; 1.2) | 0.16 | 0.47 |
| **Overall plant-based diet index** (energy adjusted) |  |  |  |  |  |  |  |
| *Model 1* | -7.0 (-12.3; -1.4) | 0.02 | 0.9 (-4.1; 6.1) | 0.73 | 1.4 (-3.1; 6.1) | 0.54 |  |
| *Model 2* | -6.8 (-12.1; -1.2) | 0.02 | 3.3 (-2.0; 8.8) | 0.23 | 3.0 (-1.4; 7.7) | 0.18 | <0.01 |
| **Healthful plant-based diet index** (energy adjusted) |  |  |  |  |  |  |  |
| *Model 1* | -10.6 (-15.6; -5.4) | <0.01 | -3.6 (-8.5; 1.7) | 0.18 | -3.1 (-7.1; 0.9) | 0.13 |  |
| *Model 2* | -8.8 (-14.0; -3.2) | <0.01 | -1.3 (-6.4; 4.0) | 0.62 | -0.5 (-4.3; 3.5) | 0.81 | 0.04 |
| **Unhealthful plant-based diet index** (energy adjusted) |  |  |  |  |  |  |  |
| *Model 1* | 5.0 (-0.8; 11.2) | 0.09 | 5.1 (-0.4; 10.8) | 0.07 | 2.6 (-1.7; 7.0) | 0.24 |  |
| *Model 2* | 1.8 (-4.2; 8.1) | 0.57 | 3.1 (-2.3; 8.7) | 0.26 | -0.1 (-4.0; 3.9) | 0.95 | 0.76 |

Regression coefficients with 95% CI for associations of dietary patterns with the cardiovascular risk score. Model 1 adjusted for age and sex, model 2 additionally adjusted for socioeconomic status, physical activity index, total daily alcohol intake (not for models including the Mediterranean diet score as exposure variable). Dietary patterns were energy adjusted using the residual method.

Regression coefficients should be interpreted as follows: relative change of the outcome variable per 1-SD increment in the exposure variable (Example: A 1-SD increment in adherence to the Mediterranean diet score (energy-adjusted), i. e. an increase by 1.61, is associated with a relative decrease in SCORE2-Diabetes by ‑6.6% (-11.7; -1.3) among SAID).

**P*-value for interaction, *P*_int_, with dietary pattern score*diabetes subtypes as interaction term calculated for the fully adjusted model 2.

1 SD of the Mediterranean diet score (energy adjusted) = 1.61; 1 SD of the DASH score (energy adjusted) = 4.79; 1 SD of the overall plant-based diet index (energy adjusted) = 6.39; 1 SD of the healthful plant-based diet index (energy adjusted) = 7.19; 1 SD of the unhealthful plant-based diet index (energy adjusted) = 7.54.

CI, confidence interval; DASH, Dietary Approaches to Stop Hypertension; MARD, mild age-related diabetes; MOD, mild obesity-related diabetes; *P*­_int_, *P*-value for interaction with dietary pattern*diabetes subtypes as interaction term; SAID, severe autoimmune diabetes; SCORE, Systematic COronary Risk Evaluation.

**Supplementary Table 3:** Associations of the food groups constituting each dietary pattern with the 10-year cardiovascular risk estimated by the Systematic COronary Risk Evaluation (SCORE)2-Diabetes stratified by diabetes subtype

| **SCORE2-Diabetes** |  | **SAID**  **(n=239)** | | **MOD**  **(n=189)** | | **MARD**  **(n=184)** | | *P*_int_^*^ |
| --- | --- | --- | --- | --- | --- | --- | --- | --- |
|  | 1 SD [g] | ß (95% CI) | *P* | ß (95% CI) | *P* | ß (95% CI) | *P* |  |
| **Mediterranean diet score** |  |  |  |  |  |  |  |  |
| **Fruit and nuts (energy adjusted)** | 144.7 |  |  |  |  |  |  |  |
| *Model 1* |  | -4.9 (-10.7; 1.2) | 0.11 | -3.4 (-8.7; 2.2) | 0.23 | -0.3 (-3.9; 3.5) | 0.89 |  |
| *Model 2* |  | -3.4 (-9.3; 3.0) | 0.29 | -0.8 (-6.3; 5.0) | 0.78 | 0.6 (-2.7; 4.0) | 0.72 | 0.38 |
| **Vegetables (energy adjusted)** | 117.5 |  |  |  |  |  |  |  |
| *Model 1* |  | -3.9 (-9.5; 2.2) | 0.20 | -2.5 (-7.2; 2.5) | 0.32 | -2.5 (-6.7; 1.8) | 0.25 |  |
| *Model 2* |  | -3.0 (-8.7; 3.1) | 0.32 | -1.0 (-5.7; 4.0) | 0.70 | -1.0 (-4.9; 3.0) | 0.61 | 0.34 |
| **Legumes (energy adjusted)** | 5.1 |  |  |  |  |  |  |  |
| *Model 1* |  | -6.9 (-12.0; -1.6) | 0.01 | -4.0 (-8.9; 1.1) | 0.12 | 1.4 (-2.9; 5.8) | 0.52 |  |
| *Model 2* |  | -6.4 (-11.5; -1.1) | 0.02 | -1.9 (-6.9; 3.3) | 0.46 | 1.6 (-2.3; 5.6) | 0.43 | 0.06 |
| **Cereals (energy adjusted)** | 91.7 |  |  |  |  |  |  |  |
| *Model 1* |  | -3.4 (-8.5; 2.0) | 0.21 | 1.3 (-3.8; 6.7) | 0.61 | -2.9 (-7.1; 1.5) | 0.19 |  |
| *Model 2* |  | -3.6 (-8.7; 1.7) | 0.18 | 0.7 (-4.2; 5.9) | 0.77 | -3.0 (-6.8; 1.0) | 0.14 | 0.35 |
| **Fish (energy adjusted)** | 22.9 |  |  |  |  |  |  |  |
| *Model 1* |  | -0.5 (-5.7; 5.0) | 0.86 | -1.7 (-7.4; 4.4) | 0.58 | -1.7 (-5.6; 2.4) | 0.41 |  |
| *Model 2* |  | 0.8 (-4.5; 6.4) | 0.77 | 0.4 (-5.4; 6.5) | 0.90 | 1.7 (-2.2; 5.6) | 0.40 | 0.94 |
| **Dairy products (energy adjusted)** | 159.7 |  |  |  |  |  |  |  |
| *Model 1* |  | -1.9 (-7.1; 3.7) | 0.50 | -0.6 (-5.9; 5.0) | 0.82 | -2.7 (-6.8; 1.5) | 0.20 |  |
| *Model 2* |  | -0.2 (-5.6; 5.5) | 0.94 | -0.8 (-5.9; 4.7) | 0.77 | -1.7 (-5.5; 2.3) | 0.40 | 0.93 |
| **Meat (energy adjusted)** | 75.4 |  |  |  |  |  |  |  |
| *Model 1* |  | 3.1 (-2.2; 8.8) | 0.26 | 1.1 (-3.9; 6.4) | 0.66 | 9.2 (4.0; 14.5) | <0.01 |  |
| *Model 2* |  | 3.0 (-2.4; 8.6) | 0.28 | 0.8 (-4.0; 5.9) | 0.74 | 5.0 (0.2; 10.1) | 0.04 | 0.46 |
| **Saturated fatty acids (energy adjusted)** | 8.8 |  |  |  |  |  |  |  |
| *Model 1* |  | -2.1 (-6.8; 2.9) | 0.41 | 0.5 (-4.9; 6.2) | 0.87 | 1.6 (-3.4; 6.9) | 0.53 |  |
| *Model 2* |  | -1.1 (-5.8; 3.8) | 0.66 | 0.8 (-4.5; 6.3) | 0.78 | 1.0 (-3.5; 5.8) | 0.66 | 0.60 |
| **Monounsaturated fatty acids (energy adjusted)** | 7.8 |  |  |  |  |  |  |  |
| *Model 1* |  | -1.7 (-6.5; 3.3) | 0.50 | -2.3 (-7.5; 3.1) | 0.40 | 1.0 (-3.9; 6.2) | 0.68 |  |
| *Model 2* |  | -0.1 (-4.9; 5.0) | 0.97 | -0.1 (-5.4; 5.5) | 0.97 | 3.1 (-1.5; 7.9) | 0.19 | 0.49 |
| **Alcohol** | 14.1 |  |  |  |  |  |  |  |
| *Model 1* |  | 0.3 (-5.3; 6.2) | 0.93 | -2.6 (-9.7; 5.0) | 0.49 | -2.6 (-6.2; 1.1) | 0.16 |  |
| *Model 2* |  | 0.9 (-4.7; 6.9) | 0.75 | -1.3 (-8.4; 6.3) | 0.73 | -1.7 (-5.0; 1.7) | 0.32 | 0.10 |
| **DASH score** |  |  |  |  |  |  |  |  |
| **Fruit (energy adjusted)** | 144.0 |  |  |  |  |  |  |  |
| *Model 1* |  | -4.2 (-10.1; 2.0) | 0.18 | -3.1 (-8.4; 2.6) | 0.28 | 0.0 (-3.6; 3.7) | 1.00 |  |
| *Model 2* |  | -2.8 (-8.7; 3.6) | 0.38 | -0.6 (-6.1; 5.2) | 0.84 | 0.6 (-2.7; 4.0) | 0.71 | 0.46 |
| **Vegetables (energy adjusted)** | 117.5 |  |  |  |  |  |  |  |
| *Model 1* |  | -3.9 (-9.5; 2.2) | 0.20 | -2.5 (-7.2; 2.5) | 0.32 | -2.5 (-6.7; 1.8) | 0.25 |  |
| *Model 2* |  | -3.0 (-8.7; 3.1) | 0.32 | -1.0 (-5.7; 4.0) | 0.70 | -1.0 (-4.9; 3.0) | 0.61 | 0.34 |
| **Nuts and legumes (energy adjusted)** | 11.4 |  |  |  |  |  |  |  |
| *Model 1* |  | -9.0 (-13.7; -4.2) | <0.01 | -7.6 (-13.2; -1.7) | 0.01 | -3.2 (-7.1; 0.7) | 0.11 |  |
| *Model 2* |  | -7.7 (-12.4; -2.7) | <0.01 | -4.8 (-10.6; 1.4) | 0.13 | 0.2 (-3.6; 4.2) | 0.90 | 0.06 |
| **Whole grain bread (energy adjusted)** | 65.6 |  |  |  |  |  |  |  |
| *Model 1* |  | -2.9 (-7.8; 2.2) | 0.26 | 0.6 (-4.8; 6.3) | 0.84 | -4.3 (-8.6; 0.2) | 0.06 |  |
| *Model 2* |  | -2.7 (-7.5; 2.3) | 0.28 | 0.6 (-4.6; 6.1) | 0.82 | -3.3 (-7.3; 0.9) | 0.12 | 0.39 |
| **Low-fat dairy (energy adjusted)** | 71.4 |  |  |  |  |  |  |  |
| *Model 1* |  | -1.0 (-5.9; 4.3) | 0.72 | -2.3 (-7.7; 3.3) | 0.40 | -2.5 (-6.8; 1.9) | 0.26 |  |
| *Model 2* |  | -0.2 (-5.1; 5.1) | 0.95 | -2.1 (-7.3; 3.3) | 0.43 | -3.0 (-6.9; 1.0) | 0.13 | 0.91 |
| **Red meat (energy adjusted)** | 53.7 |  |  |  |  |  |  |  |
| *Model 1* |  | 1.9 (-3.7; 7.8) | 0.52 | 1.0 (-3.8; 6.1) | 0.68 | 7.6 (2.8; 12.7) | <0.01 |  |
| *Model 2* |  | 1.4 (-4.1; 7.3) | 0.61 | 0.9 (-3.8; 5.8) | 0.70 | 4.4 (-0.1; 9.1) | 0.06 | 0.64 |
| **Sugar-sweetened beverages (energy adjusted)** | 348.5 |  |  |  |  |  |  |  |
| *Model 1* |  | 1.0 (-4.9; 7.1) | 0.75 | -0.3 (-4.0; 3.6) | 0.87 | 7.0 (-3.2; 18.2) | 0.19 |  |
| *Model 2* |  | -0.5 (-6.2; 5.5) | 0.86 | -1.2 (-4.8; 2.5) | 0.51 | 4.8 (-4.4; 14.9) | 0.31 | 0.54 |
| **Sodium (energy adjusted)** | 0.4 |  |  |  |  |  |  |  |
| *Model 1* |  | 0.1 (-5.2; 5.7) | 0.97 | 1.5 (-3.6; 6.9) | 0.57 | 2.5 (-2.2; 7.4) | 0.30 |  |
| *Model 2* |  | 0.9 (-4.3; 6.4) | 0.74 | 1.2 (-3.7; 6.3) | 0.64 | 0.2 (-4.0; 4.6) | 0.92 | 0.78 |
| **Plant-based diet index** |  |  |  |  |  |  |  |  |
| **Whole grain bread (energy adjusted)** | 65.6 |  |  |  |  |  |  |  |
| *Model 1* |  | -3.8 (-8.7; 1.2) | 0.14 | 0.3 (-5; 6) | 0.90 | -4.1 (-8.4; 0.5) | 0.08 |  |
| *Model 2* |  | -3.5 (-8.2; 1.5) | 0.17 | 0.4 (-4.8; 5.9) | 0.88 | -3.3 (-7.3; 0.9) | 0.12 | 0.34 |
| **Fruit (energy adjusted)** | 144.0 |  |  |  |  |  |  |  |
| *Model 1* |  | -4.2 (-10.1; 2) | 0.18 | -3.1 (-8.4; 2.6) | 0.28 | 0 (-3.6; 3.7) | 1.00 |  |
| *Model 2* |  | -2.8 (-8.7; 3.6) | 0.38 | -0.6 (-6.1; 5.2) | 0.84 | 0.6 (-2.7; 4) | 0.71 | 0.46 |
| **Vegetables (energy adjusted)** | 117.5 |  |  |  |  |  |  |  |
| *Model 1* |  | -3.9 (-9.5; 2.2) | 0.20 | -2.5 (-7.2; 2.5) | 0.32 | -2.5 (-6.7; 1.8) | 0.25 |  |
| *Model 2* |  | -3.0 (-8.7; 3.1) | 0.32 | -1 (-5.7; 4) | 0.70 | -1.0 (-4.9; 3.0) | 0.61 | 0.34 |
| **Nuts (energy adjusted)** | 9.4 |  |  |  |  |  |  |  |
| *Model 1* |  | -7.3 (-11.9; -2.4) | <0.01 | -8.0 (-14.3; -1.2) | 0.02 | -4.5 (-8.2; -0.7) | 0.02 |  |
| *Model 2* |  | -5.9 (-10.6; -0.9) | 0.02 | -5.4 (-11.9; 1.6) | 0.13 | -0.6 (-4.4; 3.4) | 0.78 | 0.29 |
| **Legumes (energy adjusted)** | 5.1 |  |  |  |  |  |  |  |
| *Model 1* |  | -6.9 (-12.0; -1.6) | 0.01 | -4.0 (-8.9; 1.1) | 0.12 | 1.4 (-2.9; 5.8) | 0.52 |  |
| *Model 2* |  | -6.4 (-11.5; -1.1) | 0.02 | -1.9 (-6.9; 3.3) | 0.46 | 1.6 (-2.3; 5.6) | 0.43 | 0.06 |
| **Vegetable oils (energy adjusted)** | 10.2 |  |  |  |  |  |  |  |
| *Model 1* |  | -1.4 (-7.1; 4.6) | 0.64 | -2 (-6.9; 3.2) | 0.45 | -2.1 (-6.1; 2.1) | 0.32 |  |
| *Model 2* |  | -0.2 (-5.9; 5.8) | 0.95 | 0.2 (-4.8; 5.4) | 0.95 | 0.3 (-3.6; 4.2) | 0.89 | 0.70 |
| **Tea and coffee (energy adjusted)** | 560.4 |  |  |  |  |  |  |  |
| *Model 1* |  | 3.7 (-2.6; 10.3) | 0.25 | 8.0 (3.1; 13.0) | <0.01 | 3.9 (-0.9; 8.9) | 0.11 |  |
| *Model 2* |  | 6.1 (-0.3; 12.8) | 0.06 | 9.6 (4.8; 14.5) | <0.01 | 4.8 (0.4; 9.3) | 0.03 | 0.41 |
| **Fruit juices (energy adjusted)** | 106.2 |  |  |  |  |  |  |  |
| *Model 1* |  | 6.2 (0.0; 12.8)^†^ | 0.05^†^ | -2.2 (-6.5; 2.4) | 0.34 | 1.4 (-3.2; 6.2) | 0.55 |  |
| *Model 2* |  | 4.5 (-1.6; 11.0) | 0.15 | -2.3 (-6.5; 2.1) | 0.30 | 1.3 (-2.9; 5.6) | 0.56 | 0.24 |
| **Refined grains (energy adjusted)** | 37.2 |  |  |  |  |  |  |  |
| *Model 1* |  | 0.9 (-3.9; 6.1) | 0.71 | 4.3 (-2.0; 11.0) | 0.18 | -0.2 (-5.1; 4.9) | 0.93 |  |
| *Model 2* |  | -0.1 (-4.9; 4.9) | 0.95 | 3.0 (-3.1; 9.4) | 0.34 | -0.3 (-4.7; 4.3) | 0.90 | 0.51 |
| **Potatoes (energy adjusted)** | 38.2 |  |  |  |  |  |  |  |
| *Model 1* |  | 2.8 (-3.1; 9.0) | 0.36 | 0.5 (-4.8; 6.0) | 0.87 | 3.6 (-0.6; 8.0) | 0.10 |  |
| *Model 2* |  | 2.1 (-3.6; 8.2) | 0.47 | 0.3 (-4.9; 5.7) | 0.92 | 1.9 (-2; 5.9) | 0.35 | 0.89 |
| **Sugar-sweetened beverages (energy adjusted)** | 348.5 |  |  |  |  |  |  |  |
| *Model 1* |  | 1.0 (-4.9; 7.1) | 0.75 | -0.3 (-4.0; 3.6) | 0.87 | 7 (-3.2; 18.2) | 0.19 |  |
| *Model 2* |  | -0.5 (-6.2; 5.5) | 0.86 | -1.2 (-4.8; 2.5) | 0.51 | 4.8 (-4.4; 14.9) | 0.31 | 0.54 |
| **Sweets and desserts (energy adjusted)** | 41.7 |  |  |  |  |  |  |  |
| *Model 1* |  | -1.3 (-6.8; 4.6) | 0.67 | 3.7 (-0.9; 8.6) | 0.12 | 2.1 (-2.9; 7.3) | 0.42 |  |
| *Model 2* |  | -0.9 (-6.4; 4.9) | 0.75 | 4.3 (-0.3; 9.0) | 0.06 | 1.5 (-3.0; 6.3) | 0.51 | 0.11 |
| **Animal fat (energy adjusted)** | 18.6 |  |  |  |  |  |  |  |
| *Model 1* |  | -0.7 (-5.8; 4.8) | 0.80 | 0.6 (-4.6; 6.2) | 0.81 | 0.0 (-4.4; 4.6) | 1.00 |  |
| *Model 2* |  | -0.4 (-5.5; 5.0) | 0.88 | -0.1 (-5.2; 5.3) | 0.98 | 0.5 (-3.6; 4.6) | 0.83 | 0.96 |
| **Dairy (energy adjusted)** | 158.4 |  |  |  |  |  |  |  |
| *Model 1* |  | -0.9 (-6.2; 4.7) | 0.75 | -0.4 (-5.8; 5.2) | 0.88 | -2.4 (-6.4; 1.9) | 0.27 |  |
| *Model 2* |  | 0.6 (-4.8; 6.2) | 0.83 | -0.8 (-6.0; 4.7) | 0.78 | -1.5 (-5.2; 2.4) | 0.45 | 0.92 |
| **Egg (energy adjusted)** | 13.4 |  |  |  |  |  |  |  |
| *Model 1* |  | 4.7 (-0.6; 10.4) | 0.08 | -1.5 (-7.1; 4.5) | 0.62 | -1.9 (-5.9; 2.2) | 0.36 |  |
| *Model 2* |  | 6.6 (1.2; 12.2) | 0.02 | -1.7 (-7.2; 4.1) | 0.55 | -1.6 (-5.2; 2.2) | 0.41 | 0.05^#^ |
| **Fish or seafood (energy adjusted)** | 28.7 |  |  |  |  |  |  |  |
| *Model 1* |  | -0.3 (-5.5; 5.2) | 0.91 | -1.5 (-7.2; 4.6) | 0.63 | -1.7 (-5.6; 2.4) | 0.41 |  |
| *Model 2* |  | 0.9 (-4.3; 6.5) | 0.73 | 0.6 (-5.2; 6.7) | 0.85 | 1.7 (-2.1; 5.7) | 0.38 | 0.95 |
| **Meat (energy adjusted)** | 75.4 |  |  |  |  |  |  |  |
| *Model 1* |  | 3.1 (-2.2; 8.8) | 0.26 | 1.1 (-3.9; 6.4) | 0.66 | 9.2 (4.0; 14.5) | <0.01 |  |
| *Model 2* |  | 3.0 (-2.4; 8.6) | 0.28 | 0.8 (-4.0; 5.9) | 0.74 | 5.0 (0.2; 10.1) | 0.04 | 0.46 |
| **Miscellaneous animal-based foods (energy adjusted)** | 4.5 |  |  |  |  |  |  |  |
| *Model 1* |  | 7.5 (1.9; 13.3) | 0.01 | -0.9 (-5.9; 4.4) | 0.73 | -3.8 (-8.8; 1.5) | 0.15 |  |
| *Model 2* |  | 5.6 (0.0; 11.4)^‡^ | 0.05^‡^ | -1.9 (-6.8; 3.1) | 0.44 | -3.4 (-7.9; 1.4) | 0.17 | 0.06 |

Regression coefficients with 95% CI for associations of the food groups constituting each dietary pattern with the cardiovascular risk score. Model 1 adjusted for age and sex, model 2 additionally adjusted for socioeconomic status, physical activity index. Food groups (except for alcohol) were energy adjusted using the residual method.

Regression coefficients should be interpreted as follows: relative change of the outcome variable per 1-SD increment in the exposure variable (Example: A 1-SD increment in legume intake (energy-adjusted), i. e. an increase by 5.1 g, is associated with a relative decrease in SCORE2-Diabetes by -6.4% (-11.5; -1.1) among SAID).

**P*-value for interaction, *P*_int_, with food group*diabetes subtypes as interaction term calculated for the fully adjusted model 2.

Effect estimates, corresponding 95% confidence intervals (CI), and *P*-values were rounded; ^†^exact *P*-value >0.05 and the 95% CI includes the null value; ^‡^exact *P*-value <0.05 and the 95% CI does not include the null value; ^#^exact *P*_int_ >0.05.

CI, confidence interval; MARD, mild age-related diabetes; MOD, mild obesity-related diabetes; SAID, severe autoimmune diabetes; SD, standard deviation; SCORE, Systematic COronary Risk Evaluation.

**Supplementary Table 4:** Associations of carbohydrate quality parameters with the 10-year cardiovascular risk estimated by the Systematic COronary Risk Evaluation (SCORE)2-Diabetes stratified by diabetes subtype

| **SCORE2-Diabetes** | **SAID**  **(n=239)** | | **MOD**  **(n=189)** | | **MARD**  **(n=184)** | | *P*_int_* |
| --- | --- | --- | --- | --- | --- | --- | --- |
|  | ß (95% CI) | *P* | ß (95% CI) | *P* | ß (95% CI) | *P* |  |
| **Dietary glycemic index** |  |  |  |  |  |  |  |
| *Model 1* | -0.1 (-5.6; 5.7) | 0.96 | 1.1 (-4.5; 7.0) | 0.71 | -2.4 (-6.5; 1.8) | 0.26 |  |
| *Model 2* | -0.4 (-5.9; 5.4) | 0.88 | -1.0 (-6.4; 4.7) | 0.73 | -4.1 (-7.8; -0.3) | 0.03 | 0.34 |
| **Dietary glycemic load** (energy adjusted) |  |  |  |  |  |  |  |
| *Model 1* | 0.4 (-4.6; 5.7) | 0.88 | 4.4 (-1.8; 11.1) | 0.17 | 0.5 (-3.5; 4.7) | 0.81 |  |
| *Model 2* | -0.3 (-5.5; 5.1) | 0.91 | 2.2 (-4.3; 9.1) | 0.51 | -2.2 (-6.0; 1.7) | 0.26 | 0.65 |
| **Low glycemic index carbohydrates^a^** (energy adjusted) |  |  |  |  |  |  |  |
| *Model 1* | -1.1 (-6.6; 4.7) | 0.71 | -3.8 (-8.6; 1.3) | 0.14 | -1.3 (-5.5; 3.0) | 0.53 |  |
| *Model 2* | -0.4 (-5.8; 5.5) | 0.90 | -2.4 (-7.2; 2.6) | 0.34 | -0.5 (-4.5; 3.7) | 0.82 | 0.82 |
| **Higher glycemic index carbohydrates^a^** (energy adjusted) |  |  |  |  |  |  |  |
| *Model 1* | 0.6 (-4.8; 6.2) | 0.84 | 6.4 (0.6; 12.5) | 0.03 | 1.5 (-2.5; 5.8) | 0.46 |  |
| *Model 2* | -0.6 (-5.9; 5.0) | 0.83 | 4.0 (-1.8; 10.2) | 0.18 | -1.2 (-4.9; 2.6) | 0.53 | 0.42 |
| **Dietary fiber** (energy adjusted) |  |  |  |  |  |  |  |
| *Model 1* | -8.7 (-13.6; -3.4) | <0.01 | -3.6 (-8.7; 1.8) | 0.18 | -4.1 (-8.0; 0.0)^†^ | 0.05^†^ |  |
| *Model 2* | -7.8 (-13.0; -2.4) | 0.01 | -1.8 (-7.3; 4.0) | 0.53 | -3.8 (-7.8; 0.4) | 0.07 | 0.07 |
| **Total sugar** (energy adjusted) |  |  |  |  |  |  |  |
| *Model 1* | -0.4 (-6.2; 5.7) | 0.89 | 0.3 (-4.4; 5.3) | 0.89 | 2.2 (-2.3; 6.9) | 0.34 |  |
| *Model 2* | -0.1 (-5.8; 6.0) | 0.98 | 0.9 (-3.8; 5.8) | 0.71 | 1.7 (-2.5; 6.0) | 0.44 | 0.53 |

Regression coefficients with 95% CI for associations of carbohydrate quality parameters with the cardiovascular risk score. Model 1 adjusted for age and sex, model 2 additionally adjusted for socioeconomic status, physical activity index, total daily alcohol intake. Dietary glycemic load, Low glycemic index and higher glycemic index carbohydrates, dietary fiber and total sugar intake were energy adjusted using the residual method.

Regression coefficients should be interpreted as follows: relative change of the outcome variable per 1-SD increment in the exposure variable (Example: A 1-SD increment in dietary fiber (energy adjusted), i. e. an increase by 6.35 g, is associated with a relative decrease in SCORE2-Diabetes -7.8% (-13.0; -2.4) among SAID).

1 SD of dietary glycemic index = 3.26; 1 SD of dietary GL (energy adjusted) = 21.78; 1 SD of low glycemic index carbohydrates (energy adjusted) = 27.15 g; 1 SD of higher glycemic index carbohydrates (energy adjusted) = 36.96 g; 1 SD of dietary fiber (energy adjusted) = 6.35 g; 1 SD of total sugar (energy adjusted) = 25.23 g.

**P*-value for interaction, *P*_int_, with carbohydrate quality parameter*diabetes subtypes as interaction term calculated for the fully adjusted model 2.

Effect estimates, corresponding 95% confidence intervals (CI), and *P*-values were rounded; ^†^exact *P*-value >0.05 and the 95% CI includes the null value.

^a^ Low glycemic index food sources are defined as ≤55. ^b^ Higher glycemic index food sources are defined as >55.

CI, confidence interval; MARD, mild age-related diabetes; MOD, mild obesity-related diabetes; SAID, severe autoimmune diabetes; SCORE, Systematic COronary Risk Evaluation.

**Supplementary Table 5:** Associations of dietary patterns with the 10-year cardiovascular risk estimated by the Systematic COronary Risk Evaluation (SCORE)2-Diabetes stratified by diabetes subtype only including individuals at baseline examination

| **SCORE2-Diabetes** | **SAID**  **(n=170)** | | **MOD**  **(n=128)** | | **MARD**  **(n=129)** | | *P*_int_* |
| --- | --- | --- | --- | --- | --- | --- | --- |
|  | ß (95% CI) | *P* | ß (95% CI) | *P* | ß (95% CI) | *P* |  |
| **Mediterranean diet score** (energy adjusted) |  |  |  |  |  |  |  |
| *Model 1* | -6.9 (-12.8; -0.6) | 0.03 | -1.8 (-7.9; 4.8) | 0.59 | -2.3 (-7.2; 2.9) | 0.37 |  |
| *Model 2* | -5.3 (-11.4; 1.3) | 0.11 | 3.7 (-2.9; 10.8) | 0.27 | 1.2 (-3.5; 6.1) | 0.62 | 0.33 |
| **DASH score** (energy adjusted) |  |  |  |  |  |  |  |
| *Model 1* | -9.5 (-15.0; -3.6) | <0.01 | -6.0 (-11.5; -0.1)^‡^ | 0.05^‡^ | -8.9 (-13.7; -3.9) | <0.01 |  |
| *Model 2* | -8.4 (-14.1; -2.2) | 0.01 | -2.4 (-8.2; 3.7) | 0.43 | -4.1 (-9.1; 1.2) | 0.13 | 0.54 |
| **Overall plant-based diet index** (energy adjusted) |  |  |  |  |  |  |  |
| *Model 1* | -8.9 (-14.9; -2.6) | 0.01 | 2.7 (-3.5; 9.4) | 0.39 | -0.4 (-5.8; 5.2) | 0.88 |  |
| *Model 2* | -8.4 (-14.3; -2.0) | 0.01 | 7.1 (0.6; 14.0) | 0.03 | 1.5 (-3.7; 7.0) | 0.58 | <0.01 |
| **Healthful plant-based diet index** (energy adjusted) |  |  |  |  |  |  |  |
| *Model 1* | -9.8 (-15.8; -3.5) | <0.01 | -3.6 (-9.6; 2.8) | 0.26 | -3.7 (-8.4; 1.2) | 0.13 |  |
| *Model 2* | -8.6 (-14.9; -1.9) | 0.01 | -0.2 (-6.4; 6.3) | 0.94 | -0.3 (-4.8; 4.5) | 0.91 | 0.23 |
| **Unhealthful plant-based diet index** (energy adjusted) |  |  |  |  |  |  |  |
| *Model 1* | 4.5 (-2.5; 12.1) | 0.21 | 7.6 (1.2; 14.4) | 0.02 | 3.5 (-1.7; 9.0) | 0.18 |  |
| *Model 2* | 1.6 (-5.6; 9.4) | 0.67 | 4.1 (-2.2; 10.7) | 0.20 | 0.3 (-4.4; 5.2) | 0.91 | 0.61 |

Regression coefficients with 95% CI for associations of dietary patterns with the cardiovascular risk score. Model 1 adjusted for age and sex, model 2 additionally adjusted for socioeconomic status, physical activity index, total daily alcohol intake (not for models including the Mediterranean diet score as exposure variable). Dietary patterns were energy adjusted using the residual method.

Regression coefficients should be interpreted as follows: relative change of the outcome variable per 1-SD increment in the exposure variable (Example: A 1-SD increment in adherence to the DASH score (energy adjusted), i. e. an increase by 4.90, is associated with a relative decrease in SCORE2-Diabetes by -8.4% (‑14.1; ‑2.2) among SAID).

1 SD of the Mediterranean diet score (energy adjusted) = 1.62; 1 SD of the DASH score (energy adjusted) = 4.90; 1 SD of the overall plant-based diet index (energy adjusted) = 6.37; 1 SD of the healthful plant-based diet index (energy adjusted) = 7.56; 1 SD of the unhealthful plant-based diet index (energy adjusted) = 7.70.

**P*-value for interaction, *P*_int_, with dietary pattern score*diabetes subtypes as interaction term calculated for the fully adjusted model 2.

Effect estimates, corresponding 95% confidence intervals (CI), and *P*-values were rounded; ^‡^exact *P*-value <0.05 and the 95% CI does not include the null value.

CI, confidence interval; MARD, mild age-related diabetes; MOD, mild obesity-related diabetes; *P*­_int_, *P*-value for interaction with dietary pattern*diabetes subtypes as interaction term; SAID, severe autoimmune diabetes; SCORE, Systematic COronary Risk Evaluation.

**Supplementary Table 6:** Associations of carbohydrate quality parameters with the 10-year cardiovascular risk estimated by the Systematic COronary Risk Evaluation (SCORE)2-Diabetes stratified by diabetes subtype only including individuals at baseline examination

| **SCORE2-Diabetes** | **SAID**  **(n=170)** | | **MOD**  **(n=128)** | | **MARD**  **(n=129)** | | *P*_int_* |
| --- | --- | --- | --- | --- | --- | --- | --- |
|  | ß (95% CI) | *P* | ß (95% CI) | *P* | ß (95% CI) | *P* |  |
| **Dietary glycemic index** |  |  |  |  |  |  |  |
| *Model 1* | 2.0 (-4.5; 9.0) | 0.55 | 4.8 (-3.1; 13.3) | 0.24 | -1.4 (-6.3; 3.6) | 0.57 |  |
| *Model 2* | 2.3 (-4.3; 9.5) | 0.50 | 0.8 (-6.7; 8.9) | 0.84 | -3.4 (-7.7; 1.2) | 0.14 | 0.16 |
| **Dietary glycemic load** (energy adjusted) |  |  |  |  |  |  |  |
| *Model 1* | 0.3 (-5.5; 6.6) | 0.91 | 8.0 (-0.5; 17.1) | 0.06 | 2.1 (-2.8; 7.3) | 0.40 |  |
| *Model 2* | 0.3 (-5.7; 6.8) | 0.92 | 3.8 (-4.5; 12.8) | 0.38 | -0.8 (-5.2; 3.9) | 0.74 | 0.64 |
| **Low glycemic index carbohydrates^a^** (energy adjusted) |  |  |  |  |  |  |  |
| *Model 1* | -6.8 (-13.0; 0.0)^‡^ | 0.05^‡^ | -4.4 (-10.1; 1.7) | 0.16 | -0.5 (-5.5; 4.7) | 0.84 |  |
| *Model 2* | -5.9 (-12.2; 0.9) | 0.09 | -1.2 (-7.0; 5.1) | 0.71 | 1.1 (-3.7; 6.1) | 0.65 | 0.05^#^ |
| **Higher glycemic index carbohydrates^a^** (energy adjusted) |  |  |  |  |  |  |  |
| *Model 1* | 3.2 (-3.3; 10.2) | 0.34 | 9.7 (2.2; 17.9) | 0.01 | 2.4 (-2.4; 7.5) | 0.32 |  |
| *Model 2* | 2.7 (-3.9; 9.8) | 0.43 | 4.5 (-3.1; 12.6) | 0.26 | -0.8 (-5.1; 3.7) | 0.71 | 0.44 |
| **Dietary fiber** (energy adjusted) |  |  |  |  |  |  |  |
| *Model 1* | -8.8 (-14.8; -2.4) | 0.01 | -3.1 (-9.1; 3.3) | 0.33 | -2.7 (-7.6; 2.4) | 0.29 |  |
| *Model 2* | -7.2 (-13.5; -0.4) | 0.04 | 0.0 (-6.5; 6.8) | 0.99 | -1.9 (-6.7; 3.1) | 0.45 | 0.09 |
| **Total sugar** (energy adjusted) |  |  |  |  |  |  |  |
| *Model 1* | -6.1 (-12.6; 0.8) | 0.08 | 0.6 (-5.4; 6.9) | 0.86 | 2.8 (-2.3; 8.3) | 0.28 |  |
| *Model 2* | -5.4 (-11.8; 1.6) | 0.13 | 1.7 (-4.1; 7.8) | 0.58 | 2.3 (-2.3; 7.2) | 0.33 | 0.02 |

Regression coefficients with 95% CI for associations of carbohydrate quality parameters with the cardiovascular risk score. Model 1 adjusted for age and sex, model 2 additionally adjusted for socioeconomic status, physical activity index, total daily alcohol intake. Dietary glycemic load, low glycemic index and higher glycemic index carbohydrates, dietary fiber and total sugar intake were energy adjusted using the residual method.

Regression coefficients should be interpreted as follows: relative change of the outcome variable per 1-SD increment in the exposure variable (Example: A 1-SD increment in dietary fiber (energy adjusted), i. e. an increase by 6.08 g, is associated with a relative decrease in the SCORE2-Diabetes by -7.2% (-13.5; -0.4) among SAID).

1 SD of dietary glycemic index = 3.27; 1 SD of dietary glycemic load (energy adjusted) = 22.05; 1 SD of low glycemic index carbohydrates (energy adjusted) = 25.21 g; 1 SD of higher glycemic index carbohydrates (energy adjusted) = 36.00 g; 1 SD of dietary fiber (energy adjusted) = 6.08 g; 1 SD of total sugar (energy adjusted) = 24.03 g.

**P*-value for interaction, *P*_int_, with carbohydrate quality parameter*diabetes subtypes as interaction term calculated for the fully adjusted model 2.

Effect estimates, corresponding 95% confidence intervals (CI), and *P*-values were rounded; ^‡^exact *P*-value <0.05 and the 95% CI does not include the null value; ^#^exact *P*_int_ <0.05.

^a^ Low glycemic index food sources are defined as ≤55. ^b^ Higher glycemic index food sources are defined as >55.

CI, confidence interval; MARD, mild age-related diabetes; MOD, mild obesity-related diabetes; SAID, severe autoimmune diabetes; SCORE, Systematic COronary Risk Evaluation.

**REFERENCES**

1. Weber KS, Schlesinger S, Goletzke J, et al. Associations of carbohydrate quality and cardiovascular risk factors vary among diabetes subtypes. *Cardiovasc Diabetol* 2025;24(1):53.

2. Goletzke J, Weber KS, Kössler T, et al. Relative validity of a glycemic index extended food-frequency questionnaire. *Nutr Metab Cardiovasc Dis* 2022;32(10):2310–20.

3. Trichopoulou A, Costacou T, Bamia C, et al. Adherence to a Mediterranean Diet and Survival in a Greek Population. *N Engl J Med* 2003;348(26):2599–608.

4. Satija A, Bhupathiraju SN, Rimm EB, et al. Plant-Based Dietary Patterns and Incidence of Type 2 Diabetes in US Men and Women: Results from Three Prospective Cohort Studies. *PLoS Med* 2016;13(6):e1002039.

5. Fung TT. Adherence to a DASH-Style Diet and Risk of Coronary Heart Disease and Stroke in Women. Arch Intern Med 2008;168(7):713.

6. Weber KS, Schlesinger S, Lang A, et al. Association of dietary patterns with diabetes-related comorbidities varies among diabetes endotypes. Nutr Metab Cardiovasc Dis 2024;34(4):911–24.
